# Supplementary material for: New insights in the targets of action of dimethyl fumarate in endothelial cells: effects on energetic metabolism and serine synthesis in vitro and in vivo
Source: Commun Biol. 2023 Oct 25;6:1084. doi: 10.1038/s42003-023-05443-4 (PMC10600195; doi:10.1038/s42003-023-05443-4)
Supplement: Supplementary file 2 — Supplementary Information [file 42003_2023_5443_MOESM2_ESM.pdf]

Supplementary Information

New insights in the targets of action of dimethyl fumarate in endothelial cells: effects on energetic metabolism and serine synthesis *in vitro* and *in vivo*

Mª Carmen Ocaña[1,2,†], Manuel Bernal[1,2,†], Chendong Yang[3,‡], Carlos Caro[4,5,‡], Alejandro Domínguez[5,‡], Hieu S. Vu[3], Casimiro Cárdenas[1,6], María Luisa Martín-García[4,5,7], Ralph J. DeBerardinis[3,8,9,10], Ana R. Quesada[1,2,11], Beatriz Martínez-Poveda[1,2,12*] and Miguel Ángel Medina[1,2,11,*]

[1] Universidad de Málaga, Andalucía Tech, Departamento de Biología Molecular y Bioquímica, Facultad de Ciencias, E-29071 Málaga, Spain

[2] IBIMA (Biomedical Research Institute of Málaga), E-29071 Málaga, Spain

[3] Children's Medical Center Research Institute, The University of Texas Southwestern Medical Center, Dallas, TX 75390, USA

[4] Biomedical Magnetic Resonance Laboratory-BMRL, Andalusian Public Foundation Progress and Health-FPS, Seville, Spain

[5] Biomedical Research Institute of Málaga and Nanomedicine Platform (IBIMA-BIONAND Platform), University of Málaga, C/Severo Ochoa, 35, 29590 Málaga, Spain

[6] Research Support Central Services (SCAI) of the University of Málaga, Spain

[7] Biomedical Research Networking Center in Bioengineering, Biomaterials & Nanomedicine (CIBER-BBN), 28029 Madrid, Spain

[8] Department of Pediatrics, The University of Texas Southwestern Medical Center, Dallas, TX 75390, USA

[9] Eugene McDermott Center for Human Growth and Development, The University of Texas Southwestern Medical Center, Dallas, TX 75390, USA

[10] Howard Hughes Medical Institute, The University of Texas Southwestern Medical Center, Dallas, TX 75390, USA

[11] CIBER de Enfermedades Raras (CIBERER), E-29071 Málaga, Spain

[12] CIBER de Enfermedades Cardiovasculares (CIBERCV), Madrid, Spain

[†] These authors contributed equally, co-first authors: Mª Carmen Ocaña, Manuel Bernal

[‡] These authors contributed equally, co-second authors: Chendong Yang, Carlos Caro, Alejandro Domínguez

[*] Correspondence: medina@uma.es; Tel.: +34 952137132; ORCID: https://orcid.org/0000-0001-7275-6462; bmpoveda@uma.es; Tel.: +34 952131674; ORCID: https://orcid.org/0000-0003-3927-3877

Supplementary Figures

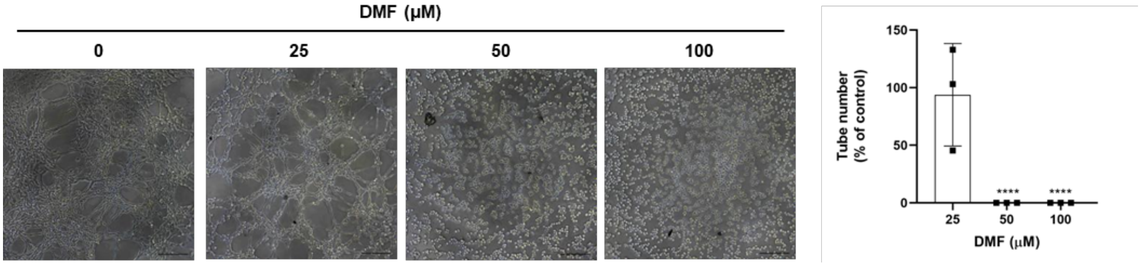

Supplementary Figure S1. DMF inhibits tube formation in HMECs. Representative pictures and quantification of tube formation on Matrigel in HMECs treated with different concentrations of DMF. Bar scales = 183.25 µm. Data are expressed as means ± SD for three independent experiments. Statistical significance was determined using the two-sided Student t-test and the resulting p-values are shown in the figure as follows: ****p<0.0001 versus untreated control.

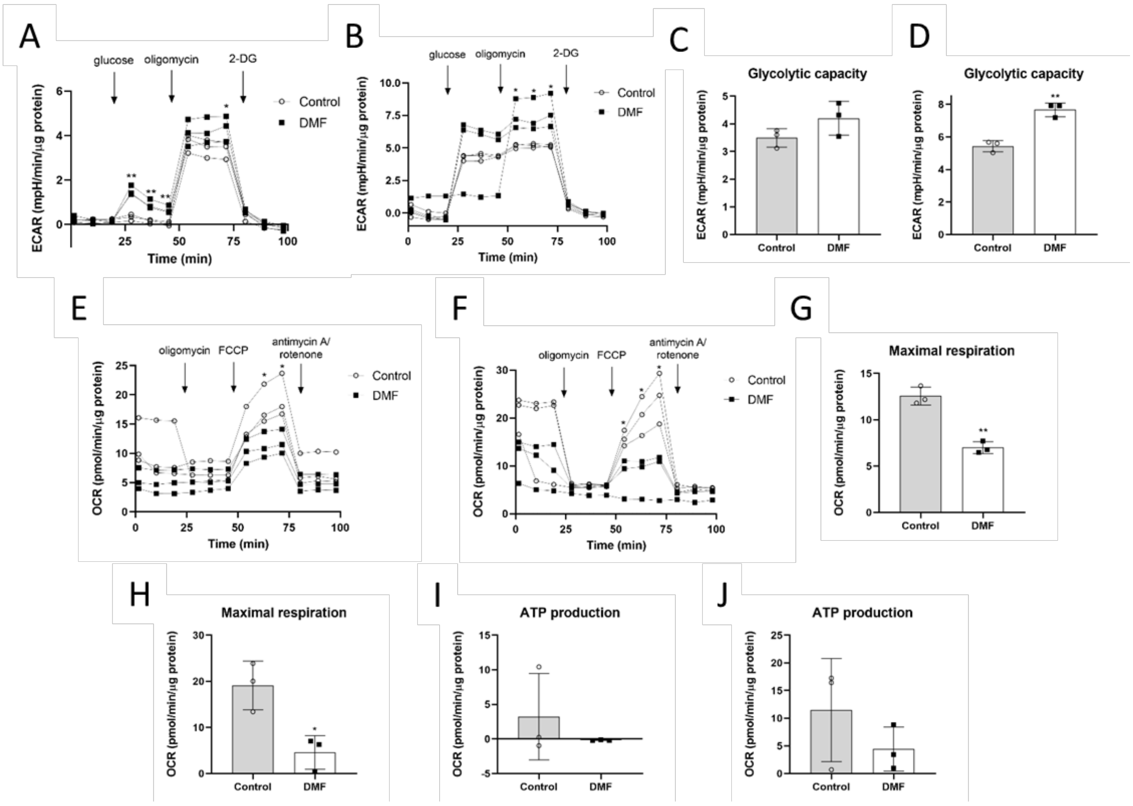

Supplementary Figure S2. DMF increases glycolysis and diminishes OXPHOS in HMECs. (A-B) ECAR was measured in cells treated with 50 µM DMF and (C-D) glycolytic capacity rate was calculated. (E-F) OCR was measured in cells treated with 50 µM DMF and (G-H) maximal respiration and (I-J) ATP production were calculated. Representative figures from individual data of three independent experiments with three replicates are shown in A-B and E-F, whereas individual data and means and SD are shown for the same experiments in C-D and G-J. Statistical significance was determined using the unpaired, two-sided Student t-test and the resulting p-values are shown in the figure as follows: *p<0.05; **p<0.01 versus untreated control.

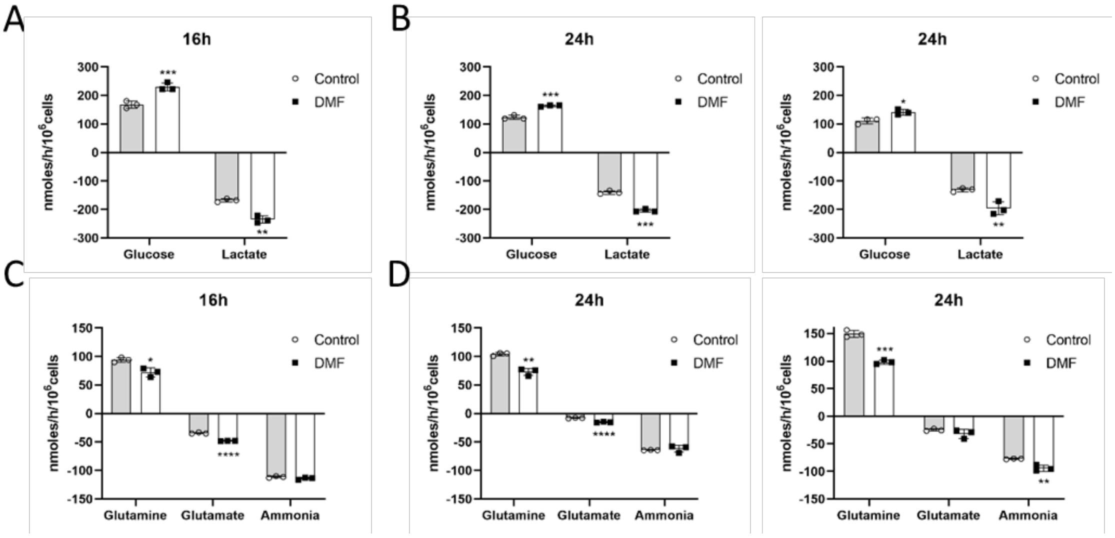

Supplementary Figure S3. Effect of DMF on glucose uptake, lactate production, glutamine uptake and glutamate and ammonia production. (A) Glucose uptake and lactate secretion in cells treated with 100 µM DMF for 16 h or (B) 24 h in medium with 10 mM glucose and 4 mM glutamine. (C) Glutamine uptake and glutamate and ammonia secretion in cells treated with 100 µM DMF for 16 h or (D) 24 h in medium with 10 mM glucose and 4 mM glutamine. Data are expressed as means ± SD of one (A and C) or two experiments (B and D) with three replicates each. Statistical significance was determined using the unpaired, two-sided Student t-test and the resulting p-values are shown in the figure as follows: *p<0.05, **p<0.01, ***p<0.001, ****p<0.0001 versus untreated control.

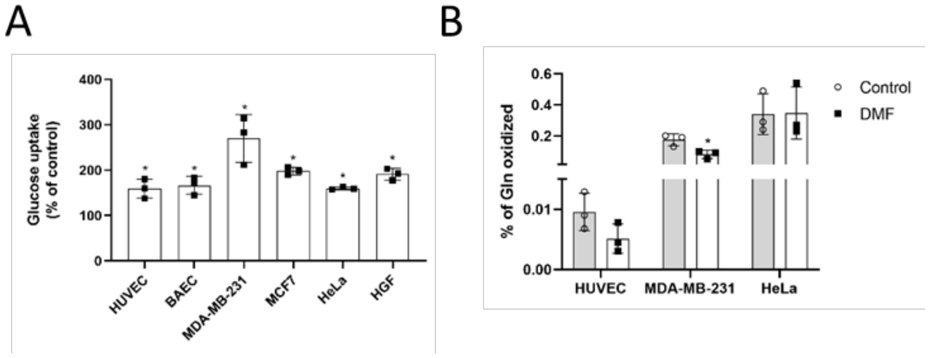

Supplementary Figure S4. Effect of DMF on glucose uptake and glutamine oxidation in other endothelial cells, tumor cells and fibroblasts. (A) Glucose uptake and (B) glutamine oxidation after 30 minutes incubation with 5 mM glucose and 0.5 mM glutamine in different cell lines treated with 100 µM DMF for 16 h. Data are expressed as means ± SD of three independent experiments. Statistical significance was determined using the unpaired, two-sided Student t-test and the resulting p-values are shown in the figure as follows: *$p<0.05$ versus untreated control.

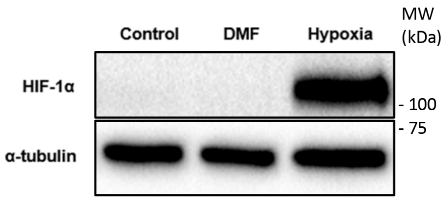

Supplementary Figure S5. Representative Western blot for HIF-1α in HMECs treated with 100 μM DMF for 24 h.

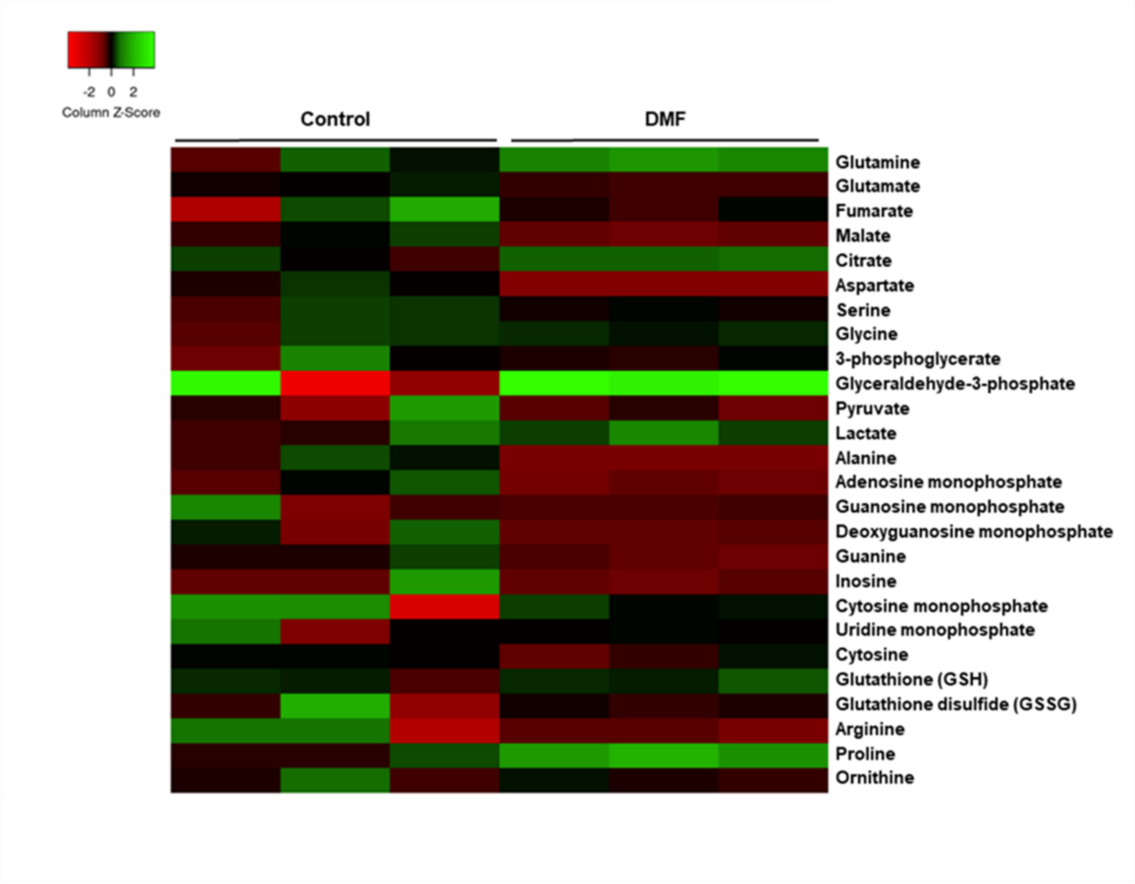

Supplementary Figure S6. Heatmap of intracellular metabolite levels in HMECs treated or not with 100 µM DMF for 24 h. Data are expressed as fold from the control condition.

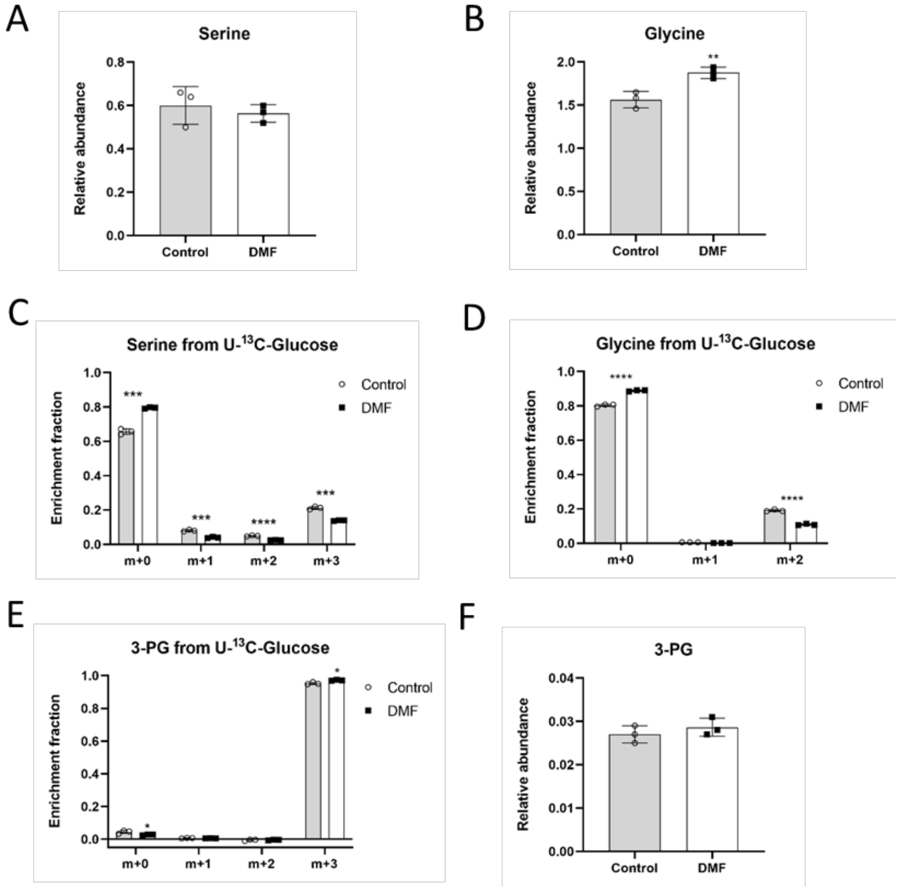

Supplementary Figure S7. Effect of 100 µM DMF overnight incubation on serine and glycine synthesis pathway in HMECs. (A) Intracellular serine and (B) glycine levels in cells treated with 100 µM DMF for 16 h. (C) Fractional labeling of serine, (D) glycine and (E) 3-PG from [U-^{13}C]-glucose and (F) intracellular 3-PG levels in cells treated with 100 µM DMF for 16 h. Data are expressed as means ± SD of one experiment with three replicates. Statistical significance was determined using the unpaired, two-sided Student t-test and the resulting p-values are shown in the figure as follows: **p<0.01, ***p<0.001, ****p<0.0001 versus untreated control.

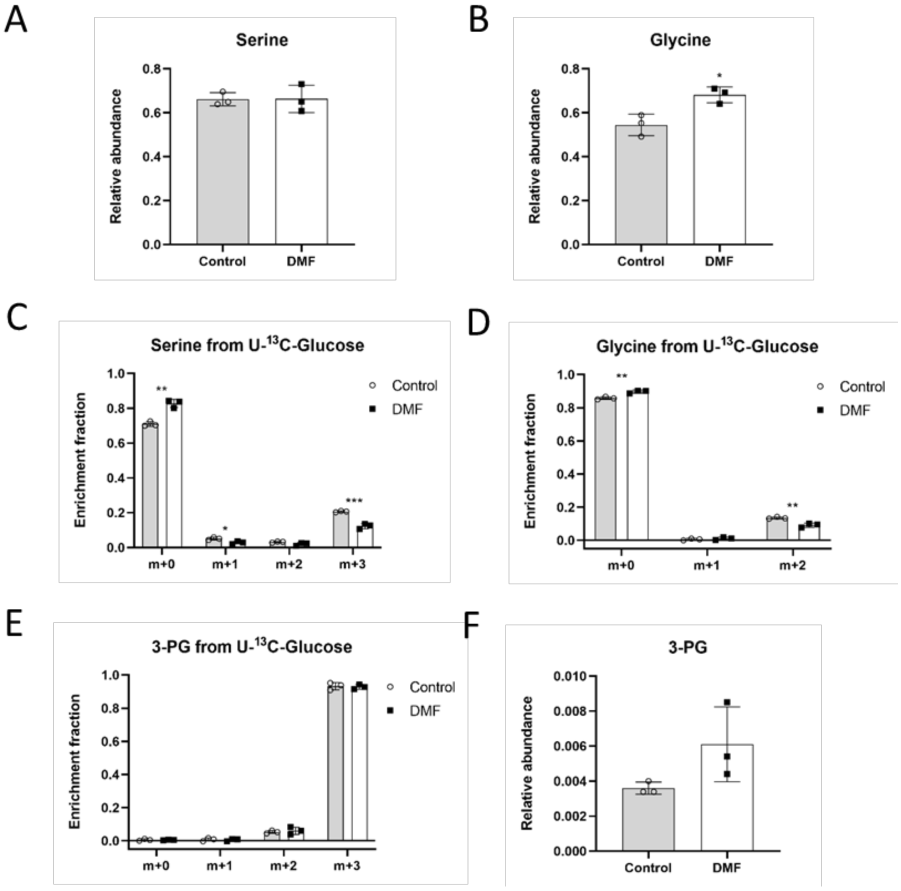

Supplementary Figure S8. Effect of 50 µM DMF for 24 h on serine and glycine synthesis pathway in HMECs. (A) Intracellular serine and (B) glycine levels in cells treated with 50 µM DMF for 24 h. (C) Fractional labeling of serine, (D) glycine and (E) 3-PG from [U-^{13}C]-glucose and (F) intracellular 3-PG levels in cells treated with 50 µM DMF for 24 h. Data are expressed as means ± SD of one experiment with three replicates. Statistical significance was determined using the unpaired, two-sided Student t-test and the resulting p-values are shown in the figure as follows: *p<0.05, **p<0.01, ***p<0.001 versus untreated control.

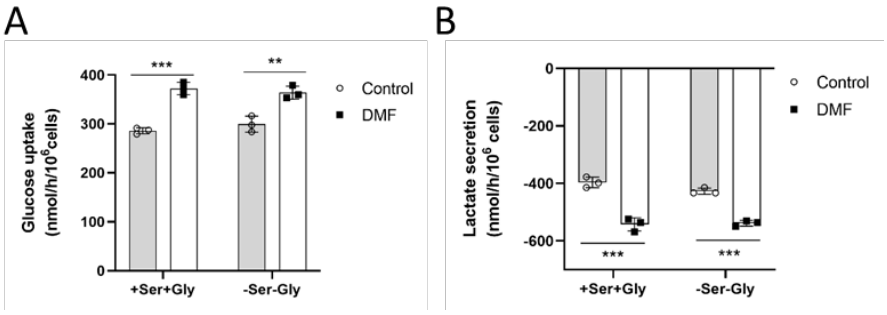

Supplementary Figure S9. Effect of DMF on glucose uptake and lactate secretion in the absence of extracellular serine and glycine. (A) Glucose uptake and (B) lactate secretion in HMECs treated with 100 µM DMF for 24 h in medium with 10 mM glucose and 4 mM glutamine in the presence or absence of 0.4 mM extracellular serine and glycine. Data are expressed as means ± SD of one experiment with three replicates. Statistical significance was determined using the unpaired, two-sided Student t-test and the resulting p-values are shown in the figure as follows: **p<0.01, ***p<0.001 versus untreated control.

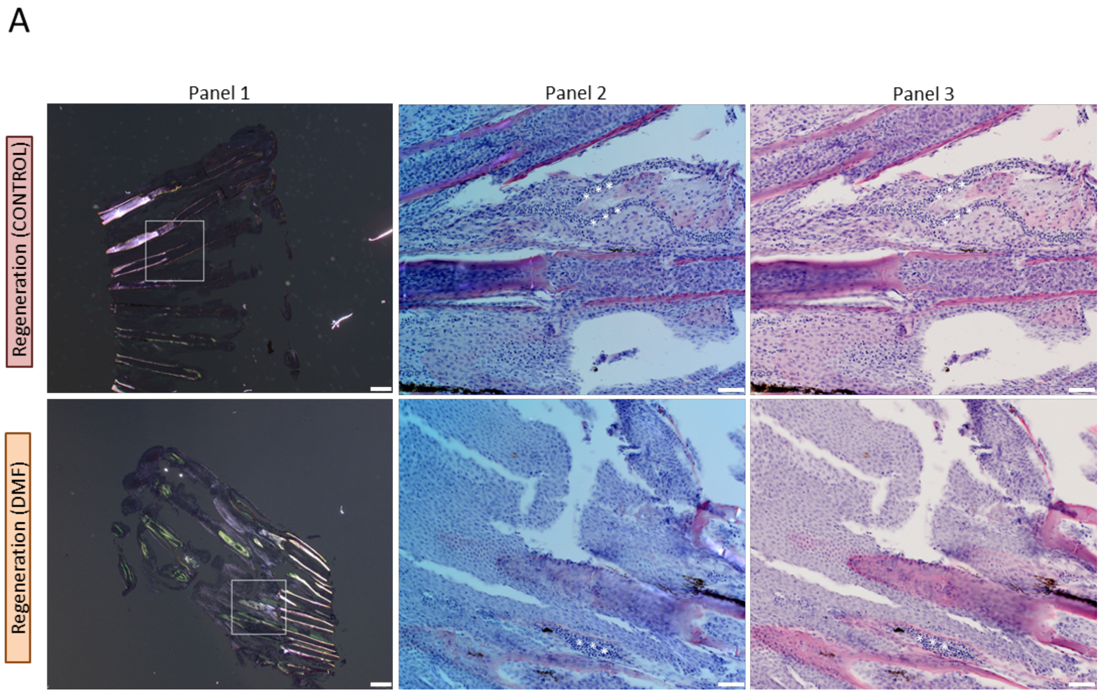

Supplementary Figure S10. Histologic analysis in the caudal fin regenerated of the zebrafish. Two regenerated conditions – with and without 25 µM DMF – were analyzed by histology. Panel 1 shows the general view of full fin stained with Picrosirius Red and polarized light at 210°; white boxes show the cropped area. Panel 2 and 3 show 10x magnification from the cropped area with polarized light at 210° and brightfield, respectively. White asterisks show the path of the blood vessels. Bar scales = 200 µm for panel 1 and 50 µm for panels 2 and 3.

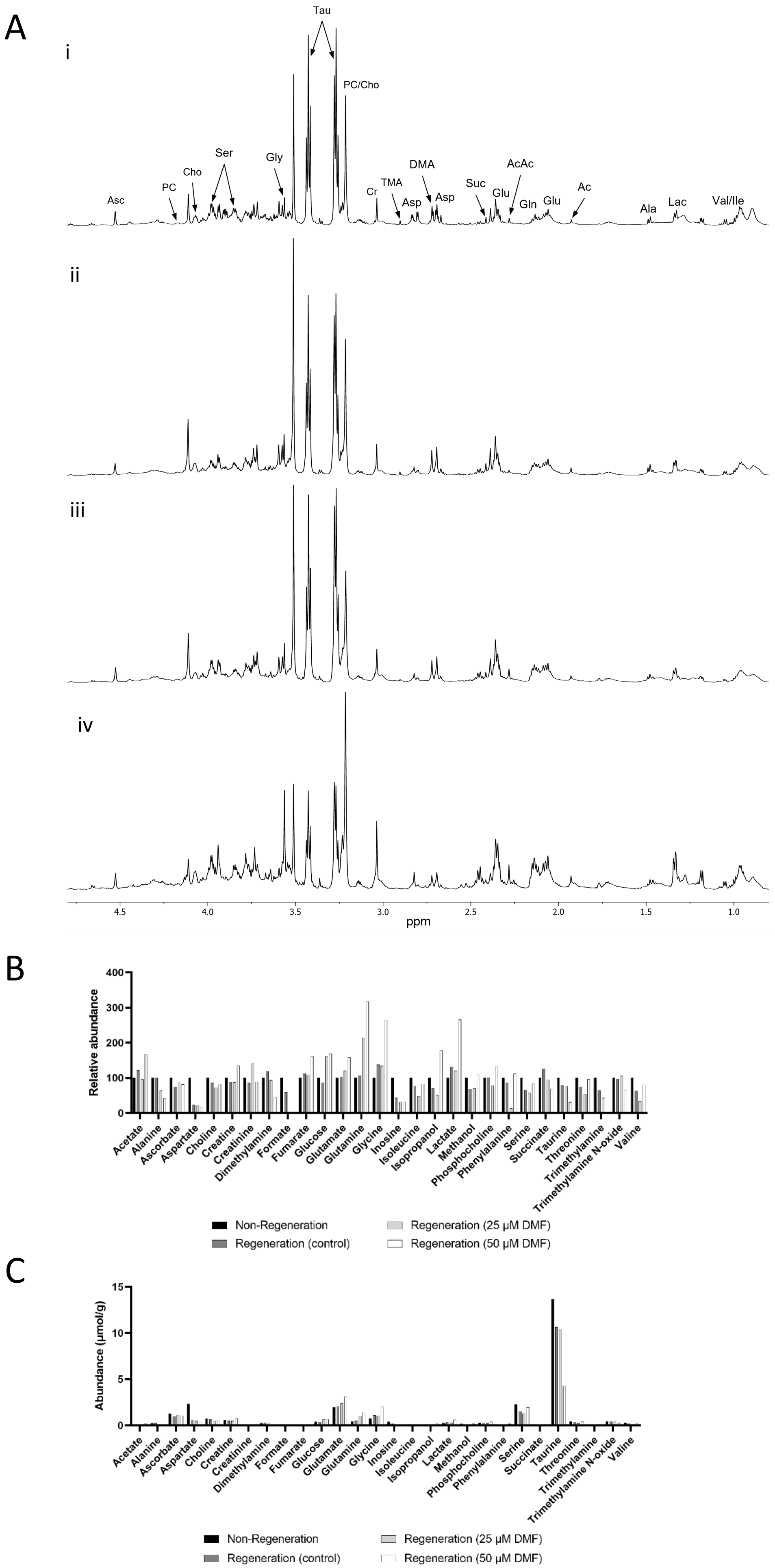

A
i
ii
iii
iv
Tau
PC/Cho
Asc
PC
Cho
Ser
Gly
Cr
TMA
Asp
DMA
Asp
Suc
Glu
AcAc
Gln
Glu
Ac
Ala
Lac
Val/Ile
4.5
4.0
3.5
3.0
2.5
2.0
1.5
1.0
ppm
B
Relative abundance
0
100
200
300
400
Acetate
Alanine
Ascorbate
Aspartate
Choline
Creatine
Creatinine
Dimethylamine
Formate
Fumarate
Glucose
Glutamate
Glutamine
Glycine
Inosine
Isoleucine
Isopropanol
Lactate
Methanol
Phosphocholine
Phenylalanine
Serine
Succinate
Taurine
Threonine
Trimethylamine
Trimethylamine N-oxide
Valine
Non-Regeneration
Regeneration (control)
Regeneration (25 µM DMF)
Regeneration (50 µM DMF)
C
Abundance (µmol/g)
0
5
10
15
Acetate
Alanine
Ascorbate
Aspartate
Choline
Creatine
Creatinine
Dimethylamine
Formate
Fumarate
Glucose
Glutamate
Glutamine
Glycine
Inosine
Isoleucine
Isopropanol
Lactate
Methanol
Phosphocholine
Phenylalanine
Serine
Succinate
Taurine
Threonine
Trimethylamine
Trimethylamine N-oxide
Valine
Non-Regeneration
Regeneration (control)
Regeneration (25 µM DMF)
Regeneration (50 µM DMF)

Supplementary Figure S11. Metabolites analyzed in the caudal fin of the zebrafish. (A) ^1H HR-MAS NMR spectra of zebrafish caudal fin. Most abundant metabolites are labeled in the upper spectra. Asc: ascorbate; PC: phosphocholine; Cho, choline; Ser: serine; Gly: glycine; Tau: taurine; Cr: creatine; TMA: trimethylamine; Asp: aspartate; DMA: dimethylamine; Suc: succinate; Glu: glutamate; AcAc: acetoacetate; Gln: glutamine; Ac: acetate; Ala: alanine; Lac: lactate; Val: valine; Ile: isoleucine. Experimental conditions were plotted as i) non-regeneration, ii) regeneration without any treatment, iii) and iv) regeneration condition treated with 25 µM and 50 µM of DMF, respectively. (B) Relative abundances were plotted compared with the non-regenerated situation for each metabolite. (C) Total abundances were plotted in µmol/g for all the metabolites compared with the internal standard.

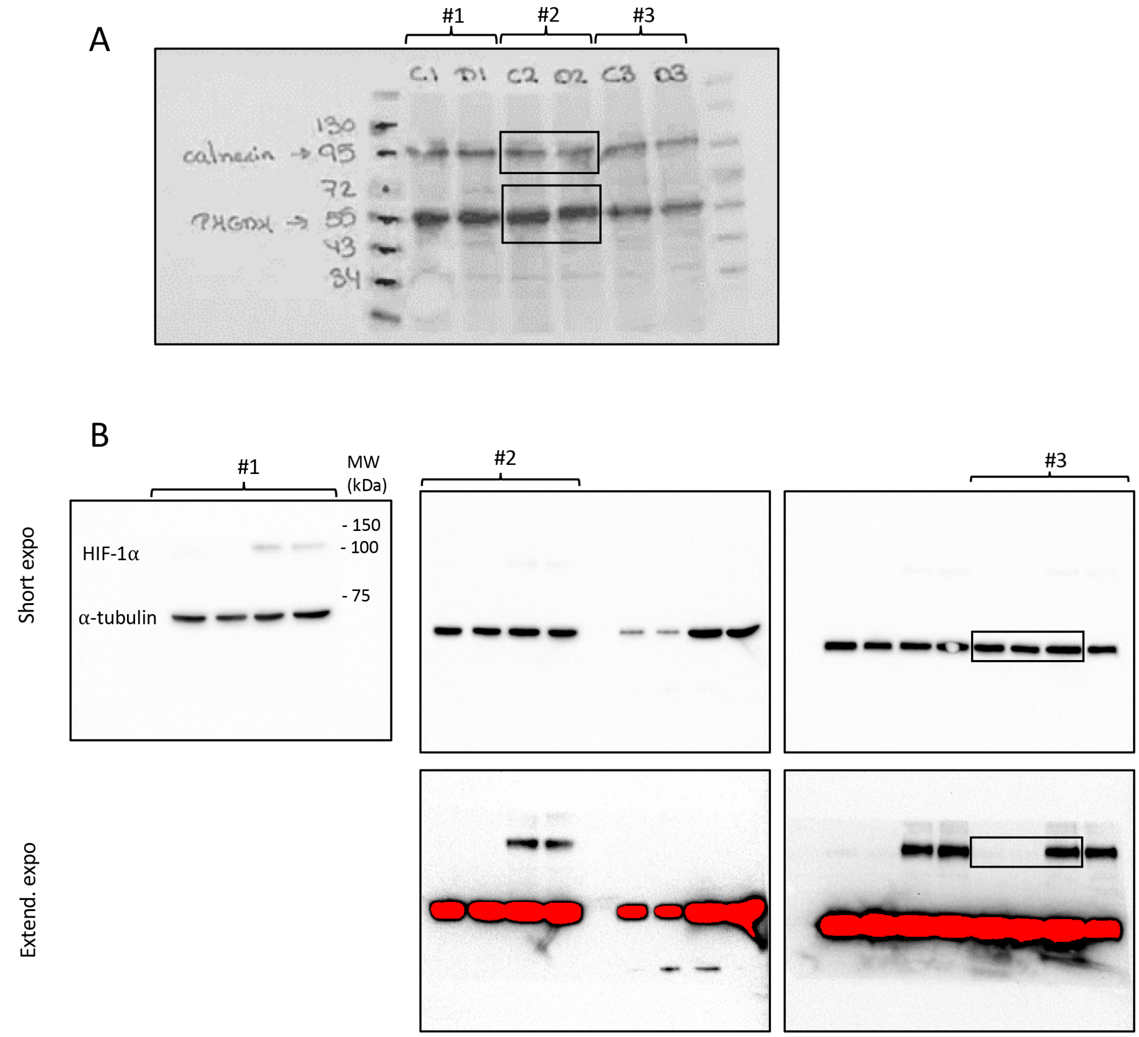

Supplementary Figure S12. (A) Uncropped immunoblot image from Figure 6 panel a; 3 independent experiments were loaded on the same gel. (B) Uncropped immunoblot images from Supplementary Figure S5; 3 independent experiments were loaded on independent gels. In both set of gels, black squares show the cropping limits used to generate de final panel

Supplementary Tables

Supplementary Table S1. Total of proteins detected with the proteomics analysis in HMECs treated with 100 µM for 24 h.

Gene Symbol	Abundance Ratio (DMF/DMSO)	P-value
**HMOX1**	26.203	1.03E-15
**GCLC**	7.12	1.03E-15
**ABHD4**	7.001	1.53E-05
**GBE1**	4.463	1.72E-11
**PSPH**	4.461	3.11E-11
**ARHGAP24**	4.429	3.81E-04
**SLC2A14**	4.063	7.13E-06
**DNM1**	3.912	3.82E-03
**TXNRD1**	3.904	2.96E-11
**SLC2A1**	3.72	7.95E-03
**RIT1**	3.701	1.03E-02
**PCYT1A**	3.526	5.65E-05
**HSPA1B; HSPA1A**	3.497	2.73E-09
**DCD**	3.446	6.15E-06
**VTN**	3.359	9.10E-04
**VWA5A**	3.236	2.94E-04
**ASNS**	3.117	2.83E-07
**RTN4**	3.107	6.39E-06
**FTH1**	3.074	2.66E-02
**SQSTM1**	3.04	5.74E-04
**ALDH1L2**	3.012	2.51E-05
**CPD**	3.008	5.90E-02
**HSPH1**	2.991	1.27E-06
**STK10**	2.991	6.03E-02
**TACC2**	2.961	1.25E-01
**AHCYL2**	2.946	1.34E-01
**BAG3**	2.932	4.46E-03
**CHORDC1**	2.765	1.16E-03
**DNAJA1**	2.76	6.39E-05
**UBE2R2**	2.76	8.45E-02
**SHANK3**	2.755	5.95E-03
**ITGA2**	2.752	8.27E-05
**ARMCX3**	2.605	1.23E-02
**CTPS1**	2.601	1.86E-02
**SARS**	2.519	6.21E-04
**CACYBP**	2.516	1.62E-03

Gene Symbol	Abundance Ratio (DMF/DMSO)	P-value
**ANTXR2**	2.516	1.72E-01
**NUFIP2**	2.512	3.03E-02
**HSPB1**	2.51	2.97E-04
**RASA1**	2.509	1.54E-01
**STAU1**	2.507	1.28E-02
**CLIP1**	2.496	8.65E-03
**CMAS**	2.485	1.14E-02
**PTGES3**	2.445	1.62E-03
**CBFB**	2.442	1.29E-01
**SLC30A1**	2.423	6.02E-02
**KLC2**	2.42	3.41E-01
**COBLL1**	2.406	2.05E-02
**CDK2**	2.393	2.49E-01
**PSAT1**	2.366	1.26E-02
**SH3PXD2B**	2.365	3.00E-01
**UAP1L1**	2.363	3.83E-02
**KPNA1**	2.361	2.34E-01
**NCDN**	2.353	2.92E-01
**CCDC71L**	2.349	8.91E-02
**TXN**	2.34	1.87E-03
**VPS28**	2.34	2.97E-01
**COTL1**	2.332	8.80E-03
**HBD**	2.324	4.52E-03
**AMFR**	2.309	3.33E-01
**FKBP5**	2.288	6.38E-03
**AMPD2**	2.288	1.91E-02
**MMP14**	2.284	2.27E-01
**MAN2A1**	2.283	2.71E-01
**SNTB2**	2.282	7.00E-03
**AIM1; CRYBG1**	2.266	1.64E-02
**RAB11FIP5**	2.264	1.13E-01
**CRYAB**	2.25	4.59E-02
**IRAK4**	2.232	3.57E-01
**SH2D3C**	2.222	3.17E-01
**SNX3**	2.189	9.15E-03
**AP1S2**	2.188	2.60E-01
**HECTD1**	2.183	8.80E-03
**TAOK1**	2.182	4.59E-01
**DNAJC7**	2.177	1.56E-01
**DNAJA2**	2.172	8.33E-02

Gene Symbol	Abundance Ratio (DMF/DMSO)	P-value
**STRBP**	2.168	1.39E-01
**TINAGL1**	2.161	4.66E-02
**EZR**	2.158	1.47E-02
**TRIP10**	2.158	3.68E-01
**EIF5**	2.156	2.04E-02
**FYCO1**	2.148	2.36E-01
**DYNC1LI2**	2.141	3.69E-01
**UAP1**	2.139	5.31E-01
**TIA1**	2.136	8.45E-02
**GNPDA2**	2.133	3.17E-01
**TPT1**	2.115	1.65E-02
**FAM160B1**	2.113	3.16E-01
**RRAGD**	2.112	2.09E-01
**NDRG1**	2.096	1.44E-01
**NEK9**	2.092	1.88E-01
**ANP32D**	2.09	2.73E-01
**TARS**	2.08	2.27E-02
**ANXA13**	2.071	5.16E-01
**ADD3**	2.069	3.12E-01
**LGALS1**	2.068	1.56E-01
**MAPRE3**	2.064	4.99E-01
**GNG2**	2.056	4.59E-01
**ME1**	2.051	7.76E-02
**ARL8A**	2.047	5.06E-01
**TOP1**	2.041	3.29E-02
**DAB2**	2.041	8.37E-02
**MAPRE2**	2.022	1.00E-01
**EEF1E1**	2.018	4.57E-01
**DNAJC3**	2.014	3.95E-01
**VAT1**	2.012	4.10E-02
**LARP4B**	2.012	5.17E-01
**RTCA**	2.01	3.97E-01
**PPIL4**	2.009	5.73E-01
**AARS**	2.008	4.27E-02
**LSM12**	2.008	3.07E-01
**HMGCS1**	2.006	6.10E-02
**CDK16**	2.001	4.91E-01
**VPS26A**	2	7.54E-02
**DDAH2**	1.984	5.75E-01
**WLS**	1.97	2.69E-01

Gene Symbol	Abundance Ratio (DMF/DMSO)	P-value
**FKBP4**	1.969	1.13E-01
**HSP90AA1**	1.956	6.71E-02
**USP47**	1.951	5.86E-01
**MON2**	1.944	1.39E-01
**NPTN**	1.94	3.78E-01
**TOP2A**	1.939	4.39E-01
**SPCS1**	1.939	5.82E-01
**DHFR; DHFRP1**	1.935	3.15E-01
**DOCK7**	1.934	4.99E-01
**TCEB1; ELOC**	1.932	2.22E-01
**LPCAT2**	1.931	4.18E-01
**ITPR1**	1.931	5.86E-01
**GSR**	1.925	1.12E-01
**YTHDF3**	1.925	3.32E-01
**ARHGAP31**	1.925	6.29E-01
**HADH**	1.925	3.27E-01
**SRSF9**	1.917	3.99E-01
**UGDH**	1.914	1.23E-01
**ARIH1**	1.912	4.11E-01
**PTPN12**	1.91	3.00E-01
**RENBP**	1.908	3.47E-01
**PSME4**	1.903	1.04E-01
**MAPK14**	1.902	1.62E-01
**PRRC2C**	1.902	2.37E-01
**EIF6**	1.89	3.16E-01
**GARS**	1.889	1.15E-01
**MAP2K1**	1.885	1.34E-01
**OS9**	1.879	6.53E-01
**SEL1L**	1.873	4.59E-01
**ARPC5L**	1.865	6.38E-01
**M6PR**	1.861	4.67E-01
**CALU**	1.854	2.14E-01
**TRIP12**	1.853	2.39E-01
**PALM2-AKAP2; PALM2; AKAP2**	1.853	3.93E-01
**ATXN2L**	1.848	2.34E-01
**TWF1**	1.847	3.42E-01
**SAR1B**	1.846	5.86E-01
**HSP90AB1**	1.843	1.54E-01
**TACC1**	1.841	6.60E-01
**CDC42**	1.84	1.87E-01

Gene Symbol	Abundance Ratio (DMF/DMSO)	P-value
**IKBIP**	1.84	3.00E-01
**SWAP70**	1.836	2.15E-01
**UBE2K**	1.831	3.77E-01
**BANF1**	1.824	3.33E-01
**ATP2C1**	1.823	5.72E-01
**DCTN3**	1.823	4.58E-01
**WDFY1**	1.823	5.26E-01
**CLPTM1**	1.82	4.18E-01
**NOVA2**	1.813	2.41E-01
**TGM2**	1.81	2.02E-01
**PTBP3**	1.809	6.76E-01
**OSBPL9**	1.808	3.77E-01
**SLC25A24**	1.806	3.77E-01
**KPNA4**	1.804	5.53E-01
**HOMER3**	1.801	4.23E-01
**TFRC**	1.801	3.61E-01
**RAB3D**	1.8	7.14E-01
**PRKD2**	1.8	7.31E-01
**HBA2; HBA1**	1.799	2.06E-01
**PACSIN2**	1.794	2.13E-01
**NRBP1**	1.794	3.61E-01
**KIF13B**	1.792	7.31E-01
**MCMBP**	1.785	7.35E-01
**RALA**	1.782	7.42E-01
**GNAQ**	1.776	4.80E-01
**FXR2**	1.772	5.97E-01
**ATP6V0A1**	1.765	3.51E-01
**LRRC8C**	1.765	4.91E-01
**GSTP1**	1.764	2.49E-01
**PGD**	1.763	2.50E-01
**CAB39**	1.762	6.97E-01
**NRD1; NRDC**	1.759	5.00E-01
**SEC13**	1.756	3.61E-01
**AK2**	1.753	5.86E-01
**ADH5**	1.753	6.31E-01
**NME1**	1.752	5.86E-01
**GSK3A**	1.752	7.43E-01
**ABCC1**	1.751	6.12E-01
**RBM39**	1.75	2.72E-01
**VPS35**	1.749	2.73E-01

Gene Symbol	Abundance Ratio (DMF/DMSO)	P-value
**WDR26**	1.749	6.64E-01
**PLCG1**	1.749	5.84E-01
**RALY**	1.749	3.16E-01
**PRPSAP1**	1.748	6.38E-01
**PACS1**	1.747	7.85E-01
**HEATR3**	1.747	7.45E-01
**MTHFD1L**	1.746	3.51E-01
**DUT**	1.746	7.18E-01
**TROVE2**	1.742	5.86E-01
**PNN**	1.741	4.78E-01
**STAT1**	1.74	2.91E-01
**BICD2**	1.738	7.67E-01
**SHC1**	1.737	5.06E-01
**LAMC1**	1.736	2.99E-01
**MRPL48**	1.735	7.92E-01
**CISD2**	1.733	5.88E-01
**HSPA8**	1.732	3.06E-01
**WDR6**	1.732	7.92E-01
**DDX6**	1.731	3.09E-01
**NUDCD2**	1.73	7.91E-01
**EPB41**	1.727	6.11E-01
**ASAH1**	1.727	7.89E-01
**DIAPH1**	1.722	3.86E-01
**LYPLA2**	1.722	6.10E-01
**S100A11**	1.719	3.61E-01
**UGP2**	1.718	3.81E-01
**RAB13**	1.716	5.75E-01
**PXN**	1.711	3.31E-01
**CLMN**	1.708	7.48E-01
**ASPH**	1.706	6.23E-01
**WBP2**	1.706	5.82E-01
**PLOD2**	1.705	4.80E-01
**G3BP2**	1.703	4.19E-01
**NUP153**	1.699	6.06E-01
**SLC9A3R2**	1.699	7.67E-01
**SRSF4**	1.698	8.05E-01
**ARID1B**	1.698	8.54E-01
**PRDX6**	1.697	3.42E-01
**SUB1**	1.697	6.10E-01
**NEDD4**	1.696	6.98E-01

Gene Symbol	Abundance Ratio (DMF/DMSO)	P-value
**ECE1**	1.695	5.98E-01
**PELO**	1.692	7.09E-01
**DCUN1D1**	1.691	8.05E-01
**TMEM33**	1.69	4.77E-01
**CASP6**	1.69	8.28E-01
**CBS; LOC102724560; CBSL**	1.688	6.11E-01
**MAOA**	1.687	5.38E-01
**LRRFIP2**	1.686	8.28E-01
**PFN1**	1.683	3.68E-01
**PPP2R4; PTPA**	1.682	5.88E-01
**GDI1**	1.681	5.31E-01
**TBCC**	1.677	8.21E-01
**TUBA1A**	1.676	5.66E-01
**SH3GLB2**	1.676	7.51E-01
**PRKAR1A**	1.674	4.50E-01
**LIMA1**	1.673	4.26E-01
**ATL2**	1.669	8.40E-01
**GPI**	1.668	4.55E-01
**ACAT2**	1.663	5.99E-01
**TJP1**	1.661	4.17E-01
**GNAI3**	1.66	8.20E-01
**UBXN7**	1.66	8.09E-01
**STXBP1**	1.659	8.79E-01
**TALDO1**	1.658	4.19E-01
**TMED1**	1.658	8.23E-01
**MOB1B**	1.657	6.98E-01
**CAPZB**	1.653	7.85E-01
**RABGAP1**	1.653	7.51E-01
**SPTBN2**	1.651	8.55E-01
**GNE**	1.651	6.38E-01
**RAB35**	1.647	6.53E-01
**KRAS**	1.647	7.63E-01
**EIF4G2**	1.645	4.49E-01
**MYO5A**	1.644	5.86E-01
**ZFR**	1.644	6.96E-01
**ANP32B**	1.642	7.25E-01
**RALB**	1.642	5.95E-01
**CPNE3**	1.639	5.51E-01
**RAI14**	1.639	5.79E-01
**LIMS1**	1.639	7.35E-01

Gene Symbol	Abundance Ratio (DMF/DMSO)	P-value
**LAMP2**	1.638	5.86E-01
**TAOK2**	1.638	8.78E-01
**EHD4**	1.637	4.67E-01
**FAM114A2**	1.636	8.32E-01
**ANKFY1**	1.633	5.86E-01
**PYCR1**	1.632	5.22E-01
**SETD7**	1.63	7.54E-01
**GLUL**	1.628	7.44E-01
**AHSA1**	1.627	5.87E-01
**ENAH**	1.627	8.18E-01
**METAP2**	1.626	7.45E-01
**LSM14A**	1.625	6.19E-01
**TCEB2; ELOB**	1.624	7.02E-01
**UCHL1**	1.623	7.19E-01
**ADARB1**	1.623	8.32E-01
**NES**	1.622	5.04E-01
**TMEM65**	1.622	8.24E-01
**ILF2**	1.62	5.08E-01
**HNRNPLL; HNRPLL**	1.62	7.94E-01
**CARS**	1.62	6.31E-01
**RBM4**	1.619	6.37E-01
**PITPNA**	1.617	7.62E-01
**ZC3H15**	1.616	7.57E-01
**SMARCC1**	1.615	8.75E-01
**RAB8B**	1.614	8.48E-01
**GALNT2**	1.612	5.86E-01
**EIF2S2**	1.611	5.29E-01
**PPAP2A; PLPP1**	1.609	7.77E-01
**CBX1**	1.608	8.32E-01
**KANK2**	1.608	8.88E-01
**STUB1**	1.607	7.90E-01
**ALDOA**	1.604	5.51E-01
**KPNA3**	1.603	7.37E-01
**CLASP1**	1.603	8.72E-01
**SKP1**	1.6	6.88E-01
**PSMA6**	1.598	5.68E-01
**MAP1B**	1.597	5.86E-01
**EIF3J**	1.597	7.04E-01
**ANXA7**	1.596	6.12E-01
**CXorf26; PBDC1**	1.595	8.75E-01

Gene Symbol	Abundance Ratio (DMF/DMSO)	P-value
**WASF2**	1.593	7.23E-01
**AAK1**	1.592	6.38E-01
**RHOG**	1.592	5.92E-01
**HEATR1**	1.592	8.02E-01
**RBM8A; LOC101060541**	1.592	8.29E-01
**ETF1**	1.591	5.80E-01
**SPTLC2**	1.59	6.11E-01
**HSPA2**	1.589	7.58E-01
**SLK**	1.588	6.68E-01
**MARS**	1.587	5.97E-01
**RTN3**	1.587	7.09E-01
**KDSR**	1.587	8.75E-01
**PRAF2**	1.587	7.98E-01
**KTN1**	1.586	5.86E-01
**ALDOC**	1.586	5.86E-01
**PHGDH**	1.586	5.86E-01
**ROCK1**	1.586	7.85E-01
**MOSPD2**	1.586	8.93E-01
**NMI**	1.586	8.82E-01
**MDH1**	1.585	5.88E-01
**REL**	1.584	8.90E-01
**YWHAG**	1.583	5.86E-01
**VASP**	1.582	7.16E-01
**ACACA**	1.582	7.99E-01
**CYB5A**	1.582	7.85E-01
**NAMPT**	1.582	8.28E-01
**DPP9**	1.582	9.13E-01
**YWHAQ**	1.581	5.86E-01
**UBE2N**	1.581	6.01E-01
**AKR1A1**	1.581	5.94E-01
**CBLB**	1.581	8.75E-01
**YME1L1**	1.58	8.90E-01
**RAB8A**	1.579	7.85E-01
**ZC3H18**	1.579	8.93E-01
**P4HA1**	1.577	6.25E-01
**S100A10**	1.577	6.67E-01
**EMC7**	1.577	8.72E-01
**VCP**	1.576	5.92E-01
**TRAM1**	1.576	8.88E-01
**NRAS**	1.574	8.72E-01

Gene Symbol	Abundance Ratio (DMF/DMSO)	P-value
**REXO2**	1.574	7.93E-01
**TNPO1**	1.573	5.98E-01
**MGEA5**	1.573	8.29E-01
**MACF1**	1.572	5.99E-01
**TRIM47**	1.572	7.77E-01
**GRB2**	1.572	8.15E-01
**HNRNPDL; HNRPDL**	1.571	7.01E-01
**PAFAH1B1**	1.57	6.10E-01
**NSF**	1.569	6.24E-01
**IST1**	1.569	7.31E-01
**ATP6V0D1**	1.569	8.01E-01
**PGK1**	1.565	6.18E-01
**UBA3**	1.565	8.67E-01
**MYH10**	1.563	9.27E-01
**TIAL1**	1.563	7.81E-01
**ERC1**	1.562	6.61E-01
**LUZP1**	1.562	8.69E-01
**RAB18**	1.56	8.05E-01
**ATG3**	1.56	8.55E-01
**ACOX1**	1.56	8.72E-01
**VPS29**	1.558	6.98E-01
**KRT14**	1.558	9.27E-01
**GOLGA5**	1.557	8.29E-01
**SPAG9**	1.556	6.38E-01
**COLGALT1; GLT25D1**	1.556	6.83E-01
**STAM**	1.556	8.82E-01
**DDX17**	1.555	6.38E-01
**DDX39A**	1.555	6.97E-01
**OLA1**	1.554	6.98E-01
**CPT1A**	1.552	8.35E-01
**WDR77**	1.552	8.50E-01
**INPPL1**	1.552	8.89E-01
**USP15**	1.55	7.78E-01
**NAGA**	1.55	7.35E-01
**PIK3C2A**	1.549	9.23E-01
**MYO1E**	1.547	8.62E-01
**GSK3B**	1.547	8.80E-01
**RHOC**	1.544	8.81E-01
**EPS15**	1.544	9.12E-01
**GNPDA1**	1.543	9.03E-01

Gene Symbol	Abundance Ratio (DMF/DMSO)	P-value
UBE2L3	1.543	8.72E-01
MIA3	1.543	8.61E-01
PYGL	1.542	7.19E-01
OXSR1	1.542	8.27E-01
OGDH	1.541	6.76E-01
BZW2	1.54	8.44E-01
ANXA4	1.538	6.82E-01
HYOU1	1.537	6.85E-01
SMAD4	1.537	9.21E-01
AK3	1.537	8.75E-01
DNAJB4	1.535	7.60E-01
DPM3	1.535	9.03E-01
SEC16A	1.535	9.08E-01
PPP1CC	1.534	9.27E-01
RAB5A	1.534	7.23E-01
C14orf166; RTRAF	1.534	7.35E-01
NIPSNAP1	1.533	9.20E-01
PPP2CB	1.531	7.27E-01
EIF4E2	1.529	9.01E-01
FLII	1.528	7.05E-01
CKAP5	1.528	7.55E-01
HSPA4	1.526	7.09E-01
API5	1.526	7.81E-01
SMCHD1	1.526	8.14E-01
EIF3G	1.525	7.31E-01
SETD3	1.525	8.82E-01
USP14	1.522	7.31E-01
BZW1	1.522	8.02E-01
LDHA	1.522	7.19E-01
HDGF	1.522	7.58E-01
NUMB	1.522	8.93E-01
HERC4	1.522	9.26E-01
TMX2	1.521	8.82E-01
TMEM160	1.521	8.85E-01
TLK1	1.519	9.27E-01
PAK2	1.518	7.31E-01
DYNLL2	1.518	9.26E-01
CD2AP	1.518	8.72E-01
TFG	1.517	7.85E-01
NAE1	1.517	8.72E-01

Gene Symbol	Abundance Ratio (DMF/DMSO)	P-value
C16orf80; CFAP20	1.517	9.28E-01
HK1	1.516	7.27E-01
KLC1	1.516	7.95E-01
TWF2	1.516	8.72E-01
PABPC1	1.515	7.30E-01
EIF3H	1.513	7.92E-01
NUP54	1.513	9.31E-01
CSDE1	1.512	7.32E-01
ALDH7A1	1.512	7.35E-01
PPP4R1	1.511	8.65E-01
UBXN1	1.511	9.28E-01
CANX	1.509	7.35E-01
DIP2B	1.509	8.21E-01
RAB3GAP2	1.508	9.31E-01
ANP32A	1.506	8.82E-01
ATP6V1E1	1.506	8.29E-01
UBE2D3	1.506	8.79E-01
ITCH	1.506	9.28E-01
FAM49B	1.505	8.75E-01
NACC1	1.505	9.28E-01
SLMAP	1.504	9.27E-01
UBA1	1.503	7.50E-01
PARK7	1.503	7.47E-01
HMGA1	1.502	8.44E-01
CYB5R3	1.501	7.55E-01
EIF4E	1.5	8.20E-01
CRK	1.5	8.51E-01
SNX27	1.5	8.72E-01
NCBP2	1.5	9.28E-01
AGFG1	1.498	9.27E-01
PPP3CA	1.497	7.62E-01
RRAGB	1.497	8.78E-01
TRA2A	1.497	8.85E-01
LBR	1.497	9.00E-01
PRMT1	1.496	7.67E-01
PSMA5	1.496	7.68E-01
PIN1	1.496	9.27E-01
ERP44	1.495	8.03E-01
WWC2	1.495	9.34E-01
ATAD3A	1.492	8.75E-01

Gene Symbol	Abundance Ratio (DMF/DMSO)	P-value
**PIGT**	1.492	9.34E-01
**NPLOC4**	1.49	8.40E-01
**PSAP**	1.49	8.75E-01
**SPTAN1**	1.489	8.23E-01
**ANXA5**	1.488	7.89E-01
**KIAA1468**	1.488	9.31E-01
**PABPC4**	1.487	8.01E-01
**CBX3; C15orf57; CCDC32**	1.487	8.97E-01
**GMPPB**	1.487	8.72E-01
**ARF5**	1.486	7.92E-01
**AHSG**	1.485	8.72E-01
**TRIM25**	1.485	9.28E-01
**SYMPK**	1.484	9.27E-01
**EXOC5**	1.484	9.28E-01
**IKBIP**	1.484	9.32E-01
**SEC23IP**	1.483	8.39E-01
**LARP1**	1.483	8.51E-01
**EEA1**	1.482	7.99E-01
**ALDH2**	1.482	7.98E-01
**EIF4G3**	1.482	8.72E-01
**AHCY**	1.481	7.98E-01
**COX6C**	1.479	8.75E-01
**SRPRB**	1.478	8.54E-01
**FKBP9**	1.478	8.79E-01
**MBNL1**	1.478	9.27E-01
**AP3S1**	1.478	9.27E-01
**ASS1**	1.477	8.02E-01
**RAB33B**	1.477	9.34E-01
**ILF3**	1.476	8.02E-01
**PPME1**	1.476	8.72E-01
**EIF2S1**	1.475	8.04E-01
**TOP2B**	1.475	8.32E-01
**THOC5**	1.475	9.34E-01
**HNRNPC**	1.474	8.05E-01
**SEC24D**	1.474	8.81E-01
**CSDA; YBX3**	1.473	8.06E-01
**IDH1**	1.473	8.05E-01
**COX7A2L**	1.473	9.31E-01
**RGPD2**	1.472	9.34E-01
**TOMM70A; TOMM70**	1.471	8.09E-01

Gene Symbol	Abundance Ratio (DMF/DMSO)	P-value
**EDF1**	1.471	9.21E-01
**COL4A3BP**	1.471	9.31E-01
**DAP3**	1.47	9.31E-01
**FGF2**	1.47	9.28E-01
**CAP1**	1.469	8.13E-01
**MARCH5**	1.469	9.34E-01
**AP1G1**	1.468	8.44E-01
**MINK1**	1.468	9.34E-01
**ATP1A1**	1.467	8.16E-01
**IARS**	1.467	8.17E-01
**UBAP2**	1.467	9.34E-01
**METAP1**	1.466	9.34E-01
**ARL8B**	1.465	9.20E-01
**PGRMC1**	1.465	9.34E-01
**ANXA2**	1.464	8.23E-01
**MLEC**	1.463	9.34E-01
**COX5A**	1.463	9.28E-01
**SCAMP3**	1.462	9.27E-01
**KIAA1715; LNPK**	1.462	9.34E-01
**FGD5**	1.462	9.34E-01
**STAT3**	1.461	8.27E-01
**YTHDF2**	1.461	9.34E-01
**ATP6V1D**	1.461	9.34E-01
**FAM175B; ABRAXAS2**	1.461	9.45E-01
**ATP6V1H**	1.459	8.72E-01
**PSME2**	1.459	8.93E-01
**RAP2B**	1.459	9.28E-01
**NCL**	1.458	8.34E-01
**DCTN4**	1.458	9.28E-01
**ARHGAP17**	1.458	9.31E-01
**PTPLAD1; HACD3**	1.458	9.19E-01
**PTPLB; HACD2**	1.458	9.37E-01
**HOOK3**	1.457	8.72E-01
**ACOT7**	1.456	8.72E-01
**PPP2R1B**	1.456	9.28E-01
**PLS3**	1.455	8.40E-01
**CAPNS1**	1.454	8.47E-01
**CDIPT**	1.454	9.34E-01
**PSMA4**	1.452	8.54E-01
**GALK1**	1.452	9.27E-01

Gene Symbol	Abundance Ratio (DMF/DMSO)	P-value
**SUPT5H**	1.451	9.28E-01
**EEF1B2**	1.45	8.50E-01
**DRG1**	1.449	8.93E-01
**DRG2**	1.449	9.22E-01
**PC**	1.448	8.54E-01
**SMARCA5**	1.448	8.54E-01
**PPA1**	1.448	8.82E-01
**CRAT**	1.448	9.28E-01
**CAPZA2**	1.446	8.82E-01
**CAMK2G**	1.446	9.34E-01
**LEMD2**	1.446	9.36E-01
**YARS**	1.445	8.63E-01
**SMG1**	1.445	9.53E-01
**RAB5B**	1.444	8.82E-01
**ITGB4**	1.444	9.50E-01
**GFPT1**	1.443	8.69E-01
**PRPS1**	1.443	9.13E-01
**PPP4C**	1.443	9.34E-01
**NUP88**	1.443	9.53E-01
**FKBP3**	1.442	9.09E-01
**GLUD1**	1.441	8.72E-01
**RAB11B**	1.441	9.52E-01
**MYEF2**	1.441	9.28E-01
**RAB21**	1.441	9.26E-01
**KIF1B**	1.441	9.53E-01
**EIF4A2**	1.44	8.93E-01
**LAMTOR1**	1.44	9.28E-01
**QKI**	1.439	9.27E-01
**EPHA2**	1.439	9.31E-01
**NCBP1**	1.439	9.31E-01
**CHCHD3**	1.439	9.28E-01
**PSMD2**	1.437	8.72E-01
**TMED7-TICAM2; TICAM2**	1.437	8.93E-01
**GLIPR2**	1.437	9.49E-01
**SLC25A4**	1.436	9.24E-01
**GSPT1**	1.436	9.18E-01
**CUL4A**	1.436	9.37E-01
**AP3D1**	1.436	9.34E-01
**STIP1**	1.435	8.75E-01
**NT5C2**	1.435	8.95E-01

Gene Symbol	Abundance Ratio (DMF/DMSO)	P-value
POR	1.435	9.34E-01
MAP1LC3A	1.435	9.34E-01
SH3GLB1	1.433	9.34E-01
CAPZB	1.432	9.53E-01
SEC62	1.432	9.53E-01
CSTF3	1.432	9.53E-01
NOP10	1.432	9.53E-01
EMC1	1.431	8.81E-01
PDCD6IP	1.43	8.82E-01
DBN1	1.43	8.82E-01
DNM2	1.429	9.22E-01
ARF4	1.429	8.82E-01
SSFA2	1.429	9.34E-01
DAPK3	1.429	9.53E-01
EIF5B	1.428	9.00E-01
PSMD14	1.428	8.84E-01
ATP6V1B2	1.427	8.83E-01
KIF2A	1.427	9.34E-01
PRKAG1	1.427	9.34E-01
PAIP1	1.427	9.53E-01
CALR	1.426	8.85E-01
ANXA1	1.426	8.90E-01
ATP5E	1.425	9.28E-01
TKT	1.424	8.88E-01
NACA	1.424	9.28E-01
CCT7	1.423	8.90E-01
PRKAR2A	1.423	9.13E-01
DCTN2	1.423	9.10E-01
TIMM44	1.423	9.33E-01
PDLIM1	1.421	8.91E-01
SPCS3	1.421	9.34E-01
SMPD4	1.421	9.53E-01
HSPD1	1.419	8.93E-01
OSTF1	1.419	9.28E-01
FAM3C; WNT16	1.419	9.53E-01
DAZAP1	1.418	9.28E-01
DDI2	1.418	9.50E-01
CCT8	1.417	8.99E-01
RBMS3	1.417	9.53E-01
PCK2	1.415	9.10E-01

Gene Symbol	Abundance Ratio (DMF/DMSO)	P-value
NOMO2	1.415	9.28E-01
AP3M1	1.415	9.34E-01
SEC22B	1.415	9.22E-01
SACM1L	1.415	9.34E-01
ARHGAP1	1.415	9.53E-01
CAMK2D	1.414	9.27E-01
MCU	1.413	9.53E-01
HSDL2	1.413	9.53E-01
SON	1.412	9.34E-01
AASDHPPT	1.412	9.43E-01
PIP4K2A	1.412	9.53E-01
PSMD11	1.411	9.12E-01
CPNE1	1.411	9.27E-01
PKM	1.409	9.13E-01
SYNCRIP	1.409	9.13E-01
YWHAH	1.409	9.13E-01
ARHGEF7	1.409	9.48E-01
ERO1L; ERO1A	1.408	9.15E-01
RHOT1	1.408	9.34E-01
PSMC5	1.407	9.18E-01
ACSL4	1.407	9.32E-01
HNRNPF	1.407	9.18E-01
PREP	1.407	9.28E-01
RCN1	1.407	9.28E-01
RAB1B	1.406	9.28E-01
RBM26	1.406	9.53E-01
APEH	1.406	9.53E-01
CCT4	1.405	9.22E-01
PLIN3	1.405	9.53E-01
STRAP	1.404	9.28E-01
UBE2V2	1.404	9.56E-01
CSNK1D	1.404	9.53E-01
MYO6	1.403	9.27E-01
EIF2S3	1.403	9.26E-01
SART3	1.403	9.34E-01
SURF4	1.403	9.28E-01
NUP214	1.402	9.53E-01
MRPL37	1.402	9.53E-01
TRIOBP	1.402	9.53E-01
YBX1	1.401	9.27E-01

Gene Symbol	Abundance Ratio (DMF/DMSO)	P-value
**AP2A2**	1.401	9.34E-01
**PSMD13**	1.401	9.27E-01
**OSBPL6**	1.401	9.53E-01
**PWP2; LOC102724159**	1.401	9.64E-01
**SSB**	1.4	9.31E-01
**PSMA3**	1.4	9.28E-01
**CORO1B**	1.4	9.34E-01
**SUMO2**	1.4	9.50E-01
**ACO2**	1.399	9.27E-01
**KPNA6**	1.399	9.60E-01
**CAT**	1.399	9.56E-01
**CCT3**	1.398	9.27E-01
**FUBP3**	1.398	9.53E-01
**GAPDH**	1.397	9.28E-01
**RAN**	1.397	9.27E-01
**PGM2**	1.397	9.28E-01
**RAB12**	1.397	9.53E-01
**SLC25A13**	1.396	9.59E-01
**A2M**	1.395	9.53E-01
**KIF5B**	1.394	9.28E-01
**CAV1**	1.394	9.28E-01
**RPL22**	1.394	9.28E-01
**CLTA**	1.394	9.31E-01
**PSMB6**	1.394	9.34E-01
**FARSA**	1.393	9.50E-01
**YTHDC1**	1.393	9.53E-01
**PSMA7**	1.392	9.28E-01
**SNX2**	1.392	9.34E-01
**YLPM1**	1.391	9.51E-01
**BCKDHB**	1.391	9.64E-01
**PAPSS1**	1.39	9.28E-01
**FSCN1**	1.389	9.28E-01
**CPSF6**	1.389	9.50E-01
**SEC63**	1.389	9.50E-01
**PALLD**	1.389	9.53E-01
**TCIRG1**	1.389	9.58E-01
**CS**	1.388	9.28E-01
**WDR82**	1.388	9.53E-01
**PSMD8**	1.388	9.46E-01
**PPP6R3**	1.388	9.53E-01

Gene Symbol	Abundance Ratio (DMF/DMSO)	P-value
**NPEPPS**	1.387	9.29E-01
**STRN3**	1.387	9.53E-01
**XPO5**	1.387	9.64E-01
**CLIC1**	1.386	9.30E-01
**EEF1G**	1.386	9.30E-01
**ARPC4**	1.386	9.30E-01
**RAC1**	1.386	9.30E-01
**GNA13**	1.385	9.53E-01
**PCNP**	1.385	9.56E-01
**NARS**	1.384	9.31E-01
**STIM1**	1.384	9.51E-01
**VPS16**	1.384	9.59E-01
**SEPT9**	1.383	9.58E-01
**YWHAZ**	1.382	9.31E-01
**EIF3I**	1.382	9.31E-01
**ATP6V1C1**	1.382	9.58E-01
**SNRPF**	1.382	9.53E-01
**PSMC2**	1.381	9.32E-01
**TCP1**	1.381	9.33E-01
**RAB10**	1.38	9.34E-01
**EPS15L1**	1.38	9.53E-01
**UBE2O**	1.38	9.75E-01
**USP7**	1.379	9.34E-01
**CUL1**	1.379	9.53E-01
**VTA1**	1.379	9.53E-01
**ESD**	1.379	9.56E-01
**SEC23A**	1.378	9.34E-01
**DDX21**	1.378	9.34E-01
**SEC61A1**	1.378	9.34E-01
**KARS**	1.378	9.34E-01
**SFXN1**	1.378	9.53E-01
**LARP7**	1.378	9.53E-01
**RSU1**	1.377	9.53E-01
**PSMB4**	1.377	9.53E-01
**SMARCD2**	1.376	9.56E-01
**VPS26B**	1.376	9.62E-01
**EDC4**	1.375	9.50E-01
**TMCO1**	1.375	9.56E-01
**SRSF6**	1.374	9.53E-01
**TMX1**	1.374	9.62E-01

Gene Symbol	Abundance Ratio (DMF/DMSO)	P-value
**PSMD6**	1.373	9.34E-01
**CFL2**	1.373	9.53E-01
**AIFM1**	1.373	9.53E-01
**SF3B14; SF3B6**	1.373	9.75E-01
**ITGA5**	1.372	9.34E-01
**NUDC**	1.372	9.53E-01
**ACAT1**	1.371	9.53E-01
**PURA**	1.371	9.54E-01
**PLAA**	1.37	9.60E-01
**NANS**	1.37	9.53E-01
**HSPA12A**	1.369	9.65E-01
**EIF3B**	1.368	9.34E-01
**RAB14**	1.368	9.34E-01
**DYNLL1**	1.368	9.67E-01
**CSNK1A1**	1.368	9.53E-01
**TNPO2**	1.367	9.73E-01
**IGF2BP2**	1.366	9.53E-01
**SNX5**	1.366	9.71E-01
**ATP5D**	1.366	9.62E-01
**TMOD3**	1.365	9.53E-01
**PPID**	1.365	9.62E-01
**XPOT**	1.365	9.56E-01
**PTGES3L-AARSD1; PTGES3L; AARSD1**	1.365	9.75E-01
**MAP2K4**	1.365	9.76E-01
**CUL4B**	1.364	9.75E-01
**PXDN**	1.364	9.53E-01
**CEP170**	1.364	9.79E-01
**CELF1**	1.363	9.53E-01
**ANP32E**	1.363	9.60E-01
**RNF2**	1.363	9.75E-01
**PSMC6**	1.362	9.48E-01
**PSMC1**	1.361	9.50E-01
**DDX5**	1.36	9.50E-01
**ROCK2**	1.36	9.53E-01
**CAPRIN1**	1.36	9.51E-01
**UBR5**	1.36	9.81E-01
**PSMB3**	1.359	9.53E-01
**SRP54**	1.359	9.61E-01
**UBA6**	1.359	9.71E-01
**SCP2**	1.356	9.83E-01

Gene Symbol	Abundance Ratio (DMF/DMSO)	P-value
**TMTC3**	1.356	9.77E-01
**CCT2**	1.354	9.53E-01
**SPCS2**	1.354	9.58E-01
**OSBPL3**	1.353	9.53E-01
**APPL1**	1.352	9.56E-01
**PDIA6**	1.352	9.53E-01
**NSFL1C**	1.352	9.64E-01
**CSNK2B**	1.352	9.64E-01
**TP53BP1**	1.352	9.53E-01
**FNDC3A**	1.351	9.75E-01
**PSMB7**	1.351	9.75E-01
**RPS5**	1.35	9.53E-01
**ABCB7**	1.349	9.84E-01
**IDH3B**	1.348	9.53E-01
**CAPN1**	1.348	9.53E-01
**SUCLG1**	1.348	9.63E-01
**PGAM4**	1.347	9.60E-01
**SAE1**	1.347	9.73E-01
**IER3IP1**	1.347	9.75E-01
**ACTN1**	1.346	9.53E-01
**UBR4**	1.346	9.53E-01
**FAM65A; RIPOR1**	1.346	9.77E-01
**ACAA2**	1.345	9.59E-01
**PPM1G**	1.345	9.75E-01
**PCMT1**	1.345	9.66E-01
**SCAMP2**	1.345	9.84E-01
**H2AFY**	1.344	9.53E-01
**PUM1**	1.344	9.64E-01
**ANO6**	1.344	9.71E-01
**ANKRD17**	1.344	9.83E-01
**TJP2**	1.343	9.53E-01
**PSMB2**	1.343	9.57E-01
**NUDT21**	1.343	9.67E-01
**TMEM214**	1.343	9.64E-01
**SEC11C**	1.343	9.83E-01
**PARL**	1.343	9.75E-01
**USO1**	1.342	9.53E-01
**SRSF1**	1.342	9.53E-01
**STOML2**	1.342	9.58E-01
**TSG101**	1.342	9.75E-01

Gene Symbol	Abundance Ratio (DMF/DMSO)	P-value
**COPG2**	1.342	9.83E-01
**OPA1**	1.341	9.53E-01
**SUGT1**	1.341	9.88E-01
**PPIH**	1.341	9.83E-01
**WDR1**	1.34	9.53E-01
**ITGB3**	1.34	9.75E-01
**VAMP3**	1.34	9.83E-01
**ATP5C1**	1.34	9.60E-01
**EHD2**	1.339	9.53E-01
**KHDRBS1**	1.339	9.53E-01
**EIF3F**	1.339	9.61E-01
**COPS6**	1.339	9.70E-01
**UBE2M**	1.339	9.75E-01
**DDX3X**	1.338	9.53E-01
**ARF6**	1.338	9.75E-01
**HGS**	1.338	9.73E-01
**MAT2B**	1.338	9.72E-01
**SEC61B**	1.338	9.72E-01
**ACTR2**	1.337	9.53E-01
**MDH2**	1.337	9.53E-01
**SEPT8**	1.337	9.83E-01
**UBXN4**	1.337	9.86E-01
**ALDH18A1**	1.336	9.53E-01
**DNM1L**	1.336	9.56E-01
**WDR12**	1.336	9.84E-01
**SET**	1.335	9.56E-01
**PSMA1**	1.335	9.55E-01
**MAP4K4**	1.335	9.83E-01
**LRRC59**	1.335	9.56E-01
**MEMO1**	1.335	9.83E-01
**HM13**	1.335	9.77E-01
**TES**	1.335	9.85E-01
**STXBP2**	1.334	9.62E-01
**TMEM43**	1.334	9.75E-01
**UPP1**	1.334	9.84E-01
**AGPAT3**	1.334	9.83E-01
**VPS33A**	1.334	9.83E-01
**KIAA0196; WASHC5**	1.333	9.83E-01
**HSP90B1**	1.332	9.57E-01
**AP3B1**	1.332	9.57E-01

Gene Symbol	Abundance Ratio (DMF/DMSO)	P-value
**TM9SF2**	1.332	9.75E-01
**PLCD1**	1.332	9.85E-01
**EIF3A**	1.331	9.58E-01
**SNRPE**	1.331	9.65E-01
**TPP2**	1.33	9.67E-01
**RBM14; RBM14-RBM4**	1.329	9.60E-01
**G6PD**	1.329	9.75E-01
**CSNK2A2**	1.329	9.79E-01
**UNC45A**	1.329	9.83E-01
**RER1**	1.329	9.86E-01
**KANK4**	1.329	9.85E-01
**PLEC**	1.328	9.83E-01
**ARCN1**	1.328	9.60E-01
**CALM3; CALM2; CALM1**	1.328	9.64E-01
**HADHA**	1.328	9.77E-01
**PPP6C**	1.328	9.87E-01
**SND1**	1.327	9.61E-01
**XPO1**	1.327	9.61E-01
**NAA15**	1.327	9.62E-01
**PPP1R7**	1.327	9.81E-01
**MESDC2; MESD**	1.327	9.86E-01
**HSPA5**	1.326	9.62E-01
**DDOST**	1.326	9.62E-01
**PRPF4B**	1.326	9.83E-01
**CPSF7**	1.326	9.84E-01
**TMX4**	1.326	9.86E-01
**PSMD7**	1.325	9.62E-01
**ACTR1A**	1.325	9.62E-01
**SUPT6H**	1.325	9.90E-01
**TRIM3**	1.325	9.84E-01
**EEF1A1**	1.323	9.62E-01
**COPG1**	1.323	9.63E-01
**EIF4G1**	1.323	9.62E-01
**EIF3CL**	1.323	9.63E-01
**PSMC4**	1.323	9.62E-01
**DDX1**	1.323	9.62E-01
**FARSB**	1.323	9.81E-01
**VAPA**	1.323	9.84E-01
**PTPN11**	1.322	9.83E-01
**PRRC2A**	1.322	9.90E-01

Gene Symbol	Abundance Ratio (DMF/DMSO)	P-value
PTPN23	1.322	9.83E-01
TTC37	1.322	9.84E-01
UBQLN1	1.321	9.83E-01
RPLP1	1.321	9.76E-01
CDC42BPB	1.321	9.83E-01
MBD2	1.321	9.85E-01
GPD1L	1.321	9.88E-01
AKR1B10	1.32	9.84E-01
HNRNPA2B1	1.319	9.67E-01
YWHAB	1.319	9.69E-01
PGLS	1.319	9.84E-01
STK24	1.319	9.86E-01
PPP2R1A	1.318	9.70E-01
GDI2	1.318	9.69E-01
GNB1	1.318	9.84E-01
RAB1A	1.318	9.84E-01
LMNA	1.317	9.72E-01
SRRT	1.317	9.79E-01
RAE1	1.317	9.95E-01
DCTN1	1.316	9.72E-01
PDIA4	1.316	9.72E-01
MAP4	1.316	9.72E-01
EEF1D	1.316	9.83E-01
CORO6	1.316	9.87E-01
GORASP2	1.316	9.87E-01
CAND1	1.315	9.73E-01
MYO1C	1.315	9.73E-01
DYNC1LI1	1.315	9.83E-01
KIAA0368	1.314	9.75E-01
SLC25A5	1.314	9.75E-01
NCKAP1	1.314	9.75E-01
SEPT11	1.314	9.84E-01
CLTC	1.313	9.75E-01
WARS	1.313	9.75E-01
PSMA2	1.313	9.83E-01
EIF4B	1.313	9.75E-01
TMED9	1.313	9.87E-01
NUCB2	1.313	9.95E-01
SLC25A22	1.313	9.88E-01
IQGAP1	1.312	9.75E-01

Gene Symbol	Abundance Ratio (DMF/DMSO)	P-value
ANXA6	1.312	9.75E-01
AP2M1	1.312	9.75E-01
NOP58	1.312	9.77E-01
FAM98A	1.312	9.88E-01
TMED2	1.312	9.83E-01
AGPS	1.312	9.88E-01
CAP2	1.312	9.87E-01
SEPT2	1.31	9.75E-01
PITPNB	1.31	9.83E-01
YES1	1.31	9.88E-01
ITGB5	1.31	9.90E-01
LYPLA1	1.31	9.90E-01
CDC37	1.309	9.83E-01
PDCD6	1.309	9.87E-01
PPA2	1.309	9.90E-01
SEC24B	1.308	9.90E-01
PLXDC2	1.308	9.88E-01
WASL; ASB15	1.308	9.98E-01
EIF3K	1.308	9.90E-01
SRGAP2	1.308	9.84E-01
PAFAH1B3	1.308	9.88E-01
ACIN1	1.307	9.88E-01
VRK1	1.307	9.90E-01
SMAP1	1.307	9.88E-01
PPFIA1	1.307	9.87E-01
HNRNPA3	1.306	9.81E-01
MLLT4; AFDN	1.306	9.83E-01
BCL2L2; PABPN1	1.306	9.87E-01
SSR4	1.306	9.83E-01
ABI1	1.305	9.88E-01
ATXN2	1.305	9.90E-01
GPHN	1.305	9.90E-01
DLD	1.304	9.83E-01
MAPK1	1.304	9.87E-01
TARDBP	1.304	9.84E-01
RAD50	1.304	9.90E-01
GRHPR	1.304	9.98E-01
UTRN	1.303	9.88E-01
SMAD2	1.303	9.87E-01
UQCRFS1P1	1.303	9.90E-01

Gene Symbol	Abundance Ratio (DMF/DMSO)	P-value
ENO1	1.302	9.83E-01
AP2S1	1.302	9.88E-01
RAP1B	1.301	9.83E-01
ETFB	1.301	9.90E-01
IMPDH1	1.301	9.88E-01
CPNE2	1.301	9.85E-01
TMEM167A	1.301	9.90E-01
NMT1	1.3	9.90E-01
RNPS1	1.3	9.90E-01
GART	1.3	9.90E-01
TPI1	1.299	9.83E-01
PSMB5	1.299	9.90E-01
SEC31A	1.298	9.83E-01
DYNC1I2	1.298	9.83E-01
HNRNPAB	1.298	9.83E-01
PSMD3	1.297	9.83E-01
VDAC2	1.297	9.83E-01
RRAS2	1.297	9.88E-01
NOP2	1.297	9.90E-01
MANF	1.297	9.90E-01
AIMP1	1.296	9.90E-01
SCAF4	1.296	1.00E+00
EPRS	1.295	9.84E-01
LGALS3	1.295	9.84E-01
PSPC1	1.295	9.90E-01
DIAPH2	1.294	9.94E-01
ACADVL	1.293	9.87E-01
HSPA9	1.292	9.84E-01
RPA1	1.292	9.95E-01
IFIT5	1.292	9.98E-01
LAMA5	1.292	9.95E-01
ACADM	1.292	9.90E-01
NXF1	1.292	9.98E-01
AP2A1	1.291	9.84E-01
RPN1	1.291	9.84E-01
NUP107	1.291	9.98E-01
HSD17B4	1.291	9.95E-01
OSBP	1.29	9.94E-01
SRSF2	1.29	9.85E-01
FAM21A; WASHC2A	1.29	9.95E-01

Gene Symbol	Abundance Ratio (DMF/DMSO)	P-value
**DHX9**	1.289	9.86E-01
**FXR1**	1.289	9.90E-01
**RPL36**	1.289	9.86E-01
**SHMT2**	1.288	9.87E-01
**ACAA1**	1.288	9.98E-01
**ILKAP**	1.288	9.90E-01
**ASNA1**	1.287	9.90E-01
**TOMM40**	1.287	9.90E-01
**COPB2**	1.286	9.87E-01
**SLC25A12**	1.286	9.87E-01
**RPL28**	1.286	9.87E-01
**NOLC1**	1.286	9.98E-01
**ST13**	1.285	9.87E-01
**TNPO3**	1.285	9.98E-01
**THUMPD1**	1.285	1.00E+00
**PLBD2**	1.285	9.98E-01
**IPO7**	1.284	9.88E-01
**CCT6A**	1.284	9.88E-01
**ADSL**	1.284	9.98E-01
**EIF4H**	1.284	9.98E-01
**LONP1**	1.283	9.88E-01
**DST**	1.283	9.90E-01
**LANCL1**	1.283	1.00E+00
**RAB2A**	1.282	9.88E-01
**GLB1**	1.282	9.90E-01
**ARHGAP18**	1.282	9.90E-01
**COPZ1**	1.281	9.98E-01
**IMPDH2**	1.28	9.92E-01
**LUC7L3**	1.28	9.95E-01
**MYCT1**	1.28	9.98E-01
**MCCC2**	1.28	9.98E-01
**EIF3L**	1.279	9.90E-01
**CCT5**	1.279	9.90E-01
**AP1M1**	1.279	9.95E-01
**CTBP1**	1.279	9.98E-01
**XPO7**	1.279	9.98E-01
**LARS**	1.278	9.92E-01
**DICER1**	1.278	9.90E-01
**COPA**	1.277	9.90E-01
**NUMA1**	1.276	9.98E-01

Gene Symbol	Abundance Ratio (DMF/DMSO)	P-value
SUCLG2	1.276	9.90E-01
YKT6	1.276	9.98E-01
BMS1	1.276	9.98E-01
DYNC1H1	1.275	9.90E-01
FERMT2	1.275	9.90E-01
IDE	1.275	9.96E-01
ADRM1	1.275	9.98E-01
SEPHS1	1.275	9.99E-01
NIN	1.275	9.98E-01
PPP1R8	1.274	9.90E-01
PANK4	1.274	9.95E-01
NAP1L1	1.273	9.90E-01
VDAC3	1.273	9.98E-01
PRRC1	1.273	9.98E-01
HINT1	1.273	9.98E-01
UBE2Z	1.273	9.98E-01
RABGGTA	1.273	9.98E-01
ALCAM	1.272	9.90E-01
UBAP2L	1.272	9.90E-01
SNX6	1.272	1.00E+00
GNL1	1.272	1.00E+00
RPS25	1.272	9.90E-01
LSM6	1.272	1.00E+00
MKL2	1.272	1.00E+00
UBA2	1.271	9.90E-01
DKC1	1.271	9.98E-01
DNAJC13	1.271	9.98E-01
PTK2	1.271	1.00E+00
C12orf23; TMEM263	1.271	9.98E-01
CSNK2A1	1.27	9.94E-01
PSMB1	1.27	1.00E+00
SEC11A	1.27	9.98E-01
CPSF1	1.27	9.95E-01
CYC1	1.269	1.00E+00
SNRNP200	1.268	9.95E-01
PSMD1	1.268	9.95E-01
VPS4B	1.268	9.98E-01
SCARB2	1.268	1.00E+00
APMAP	1.268	9.95E-01
ATP2A2	1.267	9.95E-01

Gene Symbol	Abundance Ratio (DMF/DMSO)	P-value
**CLTB**	1.267	9.98E-01
**ATL3**	1.266	9.98E-01
**LETM1**	1.266	9.98E-01
**FAM114A1**	1.266	9.98E-01
**WDR75**	1.266	9.98E-01
**TXLNA**	1.266	9.98E-01
**PEA15**	1.265	1.00E+00
**NCOA5**	1.264	9.98E-01
**COPS5**	1.264	1.00E+00
**GOT1**	1.264	9.98E-01
**PLEC**	1.263	9.98E-01
**UPF1**	1.263	9.98E-01
**DHX15**	1.263	9.98E-01
**FIP1L1**	1.263	9.98E-01
**ARPC2**	1.262	9.98E-01
**AFG3L2**	1.262	1.00E+00
**APP**	1.262	1.00E+00
**MAP2K3**	1.262	9.98E-01
**LRRC40**	1.262	9.98E-01
**USP39**	1.262	9.98E-01
**SPTAN1**	1.261	9.98E-01
**TUBB4A**	1.261	9.98E-01
**ATP5B**	1.261	9.98E-01
**PDHA1**	1.261	9.98E-01
**SUPT16H**	1.261	9.98E-01
**SH3GL1**	1.261	1.00E+00
**UCHL5**	1.261	9.98E-01
**DPYSL2**	1.26	9.98E-01
**KHSRP**	1.26	9.98E-01
**SNRPD2**	1.26	9.98E-01
**BAX**	1.26	9.98E-01
**DERA**	1.26	9.98E-01
**ARPC5**	1.26	9.98E-01
**RQCD1; CNOT9**	1.26	9.98E-01
**LAMA4**	1.259	1.00E+00
**IDH3A**	1.259	9.98E-01
**ATP5F1**	1.259	1.00E+00
**GNS**	1.258	9.98E-01
**MACROD1**	1.258	9.98E-01
**PSMD12**	1.257	9.98E-01

Gene Symbol	Abundance Ratio (DMF/DMSO)	P-value
**RPS12**	1.257	9.98E-01
**DDX39B**	1.256	1.00E+00
**NME1-NME2; NME1; NME2**	1.256	9.98E-01
**ADSS**	1.256	1.00E+00
**MTAP**	1.256	9.97E-01
**NNT**	1.255	9.99E-01
**PRDX4**	1.255	9.99E-01
**ARHGDIA**	1.254	1.00E+00
**SSR1**	1.254	9.98E-01
**XAB2**	1.253	9.90E-01
**IDH2**	1.252	1.00E+00
**NUP155**	1.252	9.91E-01
**OXCT1**	1.252	9.98E-01
**RPRD1B**	1.252	9.95E-01
**FBXO30**	1.252	9.90E-01
**PDHX**	1.252	9.98E-01
**RPL11**	1.252	1.00E+00
**HDGFRP2; HDGFL2**	1.252	9.99E-01
**HS1BP3**	1.252	9.96E-01
**VDAC1**	1.251	1.00E+00
**PTRF; CAVIN1**	1.251	1.00E+00
**ABCF2**	1.251	9.94E-01
**UBLCP1**	1.251	1.00E+00
**NHP2L1; SNU13**	1.25	9.98E-01
**CD44**	1.25	9.95E-01
**PRPF8**	1.249	1.00E+00
**GMPS**	1.248	9.99E-01
**RAB5C**	1.248	9.98E-01
**PRPF3**	1.248	9.98E-01
**SMARCA4**	1.248	9.98E-01
**AASS**	1.248	9.90E-01
**CMPK1**	1.248	9.90E-01
**SEPT7**	1.247	9.99E-01
**CKAP4**	1.247	9.99E-01
**HNRNPUL1**	1.247	9.90E-01
**SNX9**	1.247	9.90E-01
**HSPBP1**	1.247	1.00E+00
**EIF4A1**	1.246	9.98E-01
**IPO5**	1.246	9.98E-01
**EIF4A3**	1.246	9.98E-01

Gene Symbol	Abundance Ratio (DMF/DMSO)	P-value
**LRPPRC**	1.246	9.98E-01
**COPS7A**	1.246	9.88E-01
**KDELC1**	1.246	9.98E-01
**DPP3**	1.245	9.90E-01
**TM9SF4**	1.245	9.90E-01
**ACSL3**	1.244	9.90E-01
**SLC12A4**	1.244	9.90E-01
**KPNB1**	1.243	9.98E-01
**STT3B**	1.243	9.92E-01
**EXOSC10**	1.243	1.00E+00
**RPS6KA3**	1.242	9.90E-01
**RRBP1**	1.242	9.98E-01
**SRSF10; LOC100996657**	1.242	9.90E-01
**EXOC2**	1.242	9.90E-01
**SUN1**	1.242	9.90E-01
**HNRNPR**	1.241	9.98E-01
**PAICS**	1.241	9.98E-01
**RBM12**	1.241	9.90E-01
**CTH**	1.241	9.87E-01
**HSD17B10**	1.24	9.90E-01
**HSP90B2P**	1.239	9.88E-01
**RBM25**	1.239	9.95E-01
**GIT2**	1.239	9.86E-01
**CNOT1**	1.238	9.98E-01
**TMED10**	1.238	9.90E-01
**PRPF19**	1.238	9.90E-01
**FKBP8**	1.238	9.88E-01
**PA2G4**	1.237	9.98E-01
**VARS; VARS2**	1.237	9.98E-01
**FBXO3**	1.237	9.90E-01
**CNOT7**	1.237	9.90E-01
**ATP5A1**	1.236	9.98E-01
**TCEA1**	1.236	9.90E-01
**CUL2**	1.236	9.90E-01
**COPS4**	1.235	9.90E-01
**CSTF2T**	1.235	9.90E-01
**RTF1**	1.235	1.00E+00
**PSMC3**	1.234	9.95E-01
**PPP1CA**	1.234	9.90E-01
**COPS2**	1.234	9.95E-01

Gene Symbol	Abundance Ratio (DMF/DMSO)	P-value
**LMAN2**	1.234	9.86E-01
**CNP**	1.234	9.98E-01
**PRSS23**	1.233	9.87E-01
**GOLGA4**	1.233	9.98E-01
**SRP72**	1.232	9.87E-01
**MAPRE1**	1.232	9.88E-01
**CCS**	1.232	9.85E-01
**TUBB2A**	1.231	9.85E-01
**COPB1**	1.231	9.90E-01
**PSMD4**	1.231	9.90E-01
**TRAP1**	1.231	9.88E-01
**TCERG1**	1.23	9.88E-01
**SLC25A3**	1.23	9.90E-01
**MRE11A; MRE11**	1.23	9.85E-01
**FYN**	1.23	9.98E-01
**ACP1**	1.23	9.87E-01
**GOLGB1**	1.229	9.84E-01
**CDK11B**	1.229	9.84E-01
**ARF3**	1.228	9.90E-01
**PLXNB2**	1.228	9.88E-01
**FLOT2**	1.227	9.88E-01
**POLR2H**	1.227	9.88E-01
**ETFA**	1.227	9.88E-01
**ARGLU1**	1.227	9.88E-01
**SRP68**	1.226	9.88E-01
**RPS19**	1.226	9.90E-01
**LSAMP**	1.226	9.87E-01
**CDK9**	1.226	9.83E-01
**GOSR1**	1.226	9.98E-01
**AHNAK**	1.225	9.90E-01
**LDHB**	1.225	9.90E-01
**PPP2R2A**	1.224	9.89E-01
**SFXN3**	1.224	9.88E-01
**DDX18**	1.224	9.84E-01
**MICU1**	1.224	9.98E-01
**YWHAE**	1.223	9.90E-01
**DLST**	1.223	9.90E-01
**PRPF4**	1.223	9.84E-01
**EXOC3**	1.223	9.83E-01
**SNRPA1**	1.222	9.84E-01

Gene Symbol	Abundance Ratio (DMF/DMSO)	P-value
**LASP1**	1.222	9.90E-01
**HSPE1**	1.222	9.90E-01
**MAGT1**	1.222	9.87E-01
**NIPSNAP3B**	1.222	9.87E-01
**UBR1**	1.222	9.98E-01
**ACTR3**	1.221	9.89E-01
**SEPT10**	1.221	9.86E-01
**RAB7A**	1.22	9.88E-01
**CTBP2**	1.22	9.84E-01
**SBDS**	1.22	9.83E-01
**CHERP**	1.22	9.84E-01
**PPP1CB**	1.219	9.87E-01
**H2AFZ**	1.219	9.88E-01
**NCLN**	1.219	9.85E-01
**NAA50**	1.219	9.84E-01
**MAP7D1**	1.219	9.84E-01
**PMPCA**	1.218	9.84E-01
**MYADM**	1.218	9.90E-01
**TPR**	1.217	9.87E-01
**EIF3D**	1.217	9.87E-01
**CAPZA1**	1.217	9.87E-01
**PGM1**	1.217	9.88E-01
**NUP160**	1.217	9.83E-01
**HIBADH**	1.217	9.83E-01
**HADHB**	1.216	9.87E-01
**PHB2**	1.215	9.87E-01
**RARS**	1.215	9.87E-01
**HPRT1**	1.215	9.83E-01
**SDHB**	1.215	9.84E-01
**ACLY**	1.214	9.86E-01
**NUP205**	1.214	9.83E-01
**LSM2**	1.214	9.83E-01
**COASY**	1.214	9.83E-01
**BIN1**	1.214	9.90E-01
**HNRNPA1**	1.212	9.85E-01
**PTBP1**	1.212	9.85E-01
**TRIM28**	1.212	9.85E-01
**ISOC1**	1.212	9.83E-01
**SAMM50**	1.212	9.77E-01
**SNX12**	1.212	9.83E-01

Gene Symbol	Abundance Ratio (DMF/DMSO)	P-value
AGO2; EIF2C2	1.212	9.81E-01
GUSB	1.212	9.83E-01
KIAA1279; KIF1BP	1.212	9.83E-01
SNX18	1.211	9.84E-01
PDXDC1; LOC102724985	1.211	9.84E-01
EIF3E	1.21	9.84E-01
ARL1	1.21	9.83E-01
HCFC1	1.209	9.79E-01
SF3A3	1.209	9.83E-01
MRPS22	1.209	9.85E-01
TBC1D15	1.209	9.86E-01
PDCD11	1.209	9.98E-01
TRA2B	1.208	9.84E-01
EML4	1.208	9.75E-01
PGAM5	1.208	9.75E-01
ME2	1.208	9.83E-01
VPS45	1.208	9.83E-01
HUWE1	1.207	9.83E-01
RPLP0	1.207	9.83E-01
NAPA	1.207	9.75E-01
ZNF326	1.207	9.75E-01
PEBP1	1.207	9.83E-01
LRRC47	1.207	9.75E-01
MYH8	1.207	9.98E-01
USP9X	1.206	9.75E-01
PSIP1	1.206	9.71E-01
CSTF1	1.206	9.88E-01
EPB41L2	1.205	9.77E-01
EMILIN1	1.204	9.79E-01
DPM1	1.204	9.79E-01
RPS8	1.203	9.83E-01
SSRP1	1.203	9.75E-01
NAP1L4	1.203	9.83E-01
EMC2	1.203	9.75E-01
UMPS	1.203	9.72E-01
GAR1	1.203	9.83E-01
ACTN4	1.202	9.83E-01
PLOD1	1.202	9.83E-01
RPS18	1.202	9.83E-01
UBA5	1.201	9.79E-01

Gene Symbol	Abundance Ratio (DMF/DMSO)	P-value
**ZADH2**	1.201	9.73E-01
**ATP2B1**	1.2	9.75E-01
**CRKL**	1.2	9.77E-01
**NUP35**	1.2	9.90E-01
**BLVRA**	1.2	9.82E-01
**EFTUD2**	1.199	9.82E-01
**SF3A2**	1.199	9.75E-01
**MYH10**	1.198	9.69E-01
**RPSA**	1.198	9.81E-01
**NOP56**	1.198	9.81E-01
**EPPK1**	1.198	9.75E-01
**DLAT**	1.197	9.79E-01
**ACOT9**	1.197	9.75E-01
**CHTOP**	1.197	9.64E-01
**TUFM**	1.196	9.77E-01
**G3BP1**	1.196	9.77E-01
**EWSR1**	1.196	9.72E-01
**CTTN**	1.196	9.77E-01
**VIM**	1.195	9.76E-01
**CSE1L**	1.195	9.76E-01
**PCNA**	1.195	9.76E-01
**ARPC1B**	1.195	9.75E-01
**DKK2**	1.195	9.75E-01
**STK3**	1.195	9.86E-01
**HSDL1**	1.195	9.86E-01
**ADNP**	1.195	9.90E-01
**CUL3**	1.194	9.60E-01
**GLYR1**	1.194	9.84E-01
**RAB27A**	1.194	9.85E-01
**SERPINH1**	1.193	9.75E-01
**MVP**	1.193	9.75E-01
**PDS5B**	1.193	9.75E-01
**RELA**	1.193	9.62E-01
**ESAM**	1.193	9.71E-01
**EPB41L3**	1.193	9.90E-01
**BPNT1**	1.193	9.62E-01
**PLRG1**	1.193	9.75E-01
**PSMD5**	1.192	9.59E-01
**PTPRE**	1.192	9.62E-01
**CDH2**	1.192	9.60E-01

Gene Symbol	Abundance Ratio (DMF/DMSO)	P-value
SDHA	1.192	9.75E-01
DHX29	1.192	9.60E-01
APEX1	1.191	9.62E-01
U2AF1; LOC102724594; U2AF1L5	1.191	9.62E-01
P4HB	1.19	9.74E-01
GNB2	1.19	9.57E-01
RPS27	1.19	9.75E-01
KLC4	1.19	9.84E-01
HTRA1	1.189	9.72E-01
SNX1	1.189	9.68E-01
SRPK2	1.189	9.64E-01
CLIC4	1.187	9.69E-01
ARSB	1.187	9.64E-01
EFTUD1; EFL1	1.187	9.85E-01
DDX46	1.186	9.60E-01
SF1	1.186	9.62E-01
MRPS23	1.186	9.84E-01
FAR2	1.186	9.64E-01
PRKCD	1.186	9.92E-01
AP2B1	1.185	9.65E-01
CYFIP1	1.185	9.65E-01
EHD1	1.185	9.61E-01
ALDH9A1	1.185	9.63E-01
PDIA3	1.184	9.64E-01
SEC23B	1.184	9.66E-01
ALDH6A1	1.184	9.64E-01
GNA11	1.184	9.62E-01
RANBP1	1.184	9.53E-01
COPS8	1.183	9.59E-01
TRNT1	1.183	9.63E-01
LAMB1	1.182	9.63E-01
PFKP	1.182	9.56E-01
PARVA	1.182	9.60E-01
ARPC3	1.182	9.64E-01
RANBP3	1.182	9.60E-01
FHL1	1.182	9.62E-01
GLOD4	1.181	9.53E-01
ATP6V1A	1.18	9.62E-01
RUVBL2	1.18	9.62E-01
AACS	1.18	9.56E-01

Gene Symbol	Abundance Ratio (DMF/DMSO)	P-value
**OTUB1**	1.179	9.60E-01
**SRRM1**	1.179	9.59E-01
**NT5DC2**	1.179	9.62E-01
**CFL1**	1.178	9.60E-01
**GNAS**	1.178	9.53E-01
**PPP2R5D**	1.178	9.57E-01
**SLC25A1**	1.178	9.53E-01
**CDV3**	1.178	9.53E-01
**NMT2**	1.177	9.83E-01
**DHRS4**	1.177	9.56E-01
**RDX**	1.176	9.58E-01
**CDC5L**	1.176	9.53E-01
**SAFB2**	1.176	9.88E-01
**PRKCA**	1.176	9.90E-01
**WDR44**	1.175	9.53E-01
**SAR1A**	1.175	9.57E-01
**DNAJC10**	1.175	9.58E-01
**MMP2**	1.175	9.83E-01
**DCAF7**	1.175	9.56E-01
**CAPN2**	1.174	9.56E-01
**GPS1**	1.174	9.54E-01
**RCC1**	1.174	9.53E-01
**PCYT2**	1.174	9.56E-01
**ARFGAP3**	1.174	9.56E-01
**QARS**	1.173	9.53E-01
**SNRPD3**	1.173	9.53E-01
**SEC24A**	1.173	9.84E-01
**TAGLN2**	1.172	9.55E-01
**LUC7L2**	1.172	9.53E-01
**NAA10**	1.172	9.53E-01
**LMAN1**	1.172	9.53E-01
**UBE4A**	1.172	9.56E-01
**POLDIP2**	1.172	9.83E-01
**RANBP2**	1.171	9.52E-01
**SRSF7**	1.171	9.53E-01
**RPN2**	1.17	9.53E-01
**AQR**	1.17	9.53E-01
**EIF2B4**	1.17	9.75E-01
**PTPN2**	1.17	9.86E-01
**FLNB**	1.169	9.53E-01

Gene Symbol	Abundance Ratio (DMF/DMSO)	P-value
HNRNPD	1.169	9.53E-01
HNRNPH3	1.169	9.53E-01
ELAVL1	1.168	9.53E-01
NUP93	1.168	9.53E-01
RAB6A	1.168	9.53E-01
ACBD3	1.168	9.53E-01
LAP3	1.168	9.53E-01
TUBA4A	1.167	9.53E-01
RPL18A	1.167	9.53E-01
FAU	1.167	9.52E-01
DYSF	1.166	9.53E-01
RPS10	1.166	9.53E-01
U2AF2	1.165	9.53E-01
PFKL	1.165	9.53E-01
PKN2	1.165	9.53E-01
WDR3	1.165	9.53E-01
SMARCC2	1.164	9.53E-01
DDX23	1.164	9.50E-01
TIPRL	1.163	9.53E-01
CDC42EP1	1.163	9.84E-01
RPL15	1.162	9.53E-01
PFAS	1.162	9.53E-01
TMEM11	1.162	9.53E-01
SUCLA2	1.161	9.53E-01
TXNL1	1.161	9.51E-01
SEC24C	1.161	9.52E-01
RASAL2	1.161	9.53E-01
CTTNBP2NL	1.161	9.53E-01
RANGAP1	1.161	9.53E-01
ERGIC3	1.161	9.58E-01
FMNL3	1.161	9.83E-01
MYO1D	1.16	9.53E-01
FH	1.16	9.53E-01
IMMT	1.16	9.53E-01
EIF3M	1.16	9.53E-01
SNRNP40	1.16	9.53E-01
CLPX	1.16	9.53E-01
PYCRL; PYCR3	1.16	9.39E-01
RAB3GAP1	1.16	9.75E-01
C22orf28; RTCB	1.159	9.53E-01

Gene Symbol	Abundance Ratio (DMF/DMSO)	P-value
**ATP2B4**	1.159	9.53E-01
**PSME3**	1.159	9.53E-01
**BAG6**	1.158	9.48E-01
**CNDP2**	1.158	9.53E-01
**THOC2**	1.157	9.53E-01
**GPAA1**	1.157	9.86E-01
**SEPT5**	1.157	9.75E-01
**PRPF40A**	1.156	9.53E-01
**PDCD5**	1.156	9.53E-01
**ANXA3**	1.156	9.53E-01
**FABP3**	1.156	9.46E-01
**IDI1**	1.156	9.53E-01
**LPP**	1.154	9.34E-01
**VAPB**	1.154	9.53E-01
**PPIC**	1.154	9.53E-01
**EXOC4**	1.154	9.75E-01
**ZNF207**	1.154	9.53E-01
**ATIC**	1.153	9.51E-01
**RPS20**	1.153	9.52E-01
**IARS2**	1.153	9.34E-01
**IK**	1.153	9.53E-01
**CRIP2**	1.153	9.34E-01
**HDLBP**	1.152	9.51E-01
**TM9SF3**	1.152	9.39E-01
**XRN2**	1.152	9.34E-01
**TANC1**	1.152	9.53E-01
**TMED4**	1.152	9.53E-01
**PPP1R18**	1.152	9.74E-01
**SEH1L**	1.151	9.53E-01
**MTCH1**	1.15	9.50E-01
**PRMT5**	1.15	9.34E-01
**SRI**	1.15	9.48E-01
**LMNB2**	1.149	9.49E-01
**FLOT1**	1.148	9.34E-01
**SRSF11**	1.148	9.53E-01
**KRT5**	1.147	9.53E-01
**BASP1**	1.147	9.34E-01
**TIMM17B**	1.147	9.83E-01
**RPS3**	1.146	9.41E-01
**ARPC1A**	1.146	9.34E-01

Gene Symbol	Abundance Ratio (DMF/DMSO)	P-value
**CNOT3**	1.146	9.50E-01
**TMEM189-UBE2V1; UBE2V1**	1.145	9.72E-01
**RHOA**	1.144	9.34E-01
**PYGB**	1.144	9.34E-01
**RPS15A**	1.144	9.34E-01
**NASP**	1.144	9.31E-01
**SLC25A10**	1.144	9.48E-01
**MATR3**	1.143	9.34E-01
**ACSS2**	1.143	9.48E-01
**PNP**	1.143	9.34E-01
**HNRNPUL2**	1.142	9.34E-01
**RPS9**	1.142	9.34E-01
**RPS15**	1.142	9.49E-01
**CHMP4B**	1.142	9.48E-01
**RHOT2**	1.142	9.83E-01
**UGGT1**	1.141	9.34E-01
**ABCF1**	1.141	9.34E-01
**RPS26; LOC101929876; RPS26P25**	1.141	9.34E-01
**EIF1AX; LOC101060318; LOC107984923**	1.141	9.50E-01
**STK38L**	1.141	9.50E-01
**CCDC22**	1.141	9.81E-01
**SPTBN1**	1.14	9.34E-01
**RPL12**	1.14	9.34E-01
**EIF5A**	1.14	9.34E-01
**CARM1**	1.14	9.30E-01
**POLR2A**	1.14	9.34E-01
**ESYT2**	1.14	9.30E-01
**MRPL39**	1.14	9.53E-01
**AP1B1**	1.139	9.34E-01
**ITGAV**	1.139	9.34E-01
**PHB**	1.139	9.34E-01
**HNRNPH2**	1.139	9.34E-01
**PFKM**	1.139	9.43E-01
**PMPCB**	1.138	9.28E-01
**COPS3**	1.137	9.28E-01
**DARS**	1.136	9.34E-01
**PEF1**	1.136	9.37E-01
**RPS2**	1.135	9.34E-01
**SAFB**	1.135	9.28E-01
**GLRX3**	1.135	9.28E-01

Gene Symbol	Abundance Ratio (DMF/DMSO)	P-value
**KAT7**	1.135	9.34E-01
**CSK**	1.135	9.34E-01
**COG4**	1.135	9.70E-01
**MRPL11**	1.135	9.75E-01
**CAD**	1.134	9.34E-01
**GAPVD1**	1.134	9.28E-01
**FAM120A**	1.134	9.28E-01
**SRSF3**	1.134	9.28E-01
**FMR1**	1.134	9.83E-01
**PGAM1; LOC643576**	1.133	9.34E-01
**RPS4X**	1.133	9.33E-01
**SF3A1**	1.133	9.32E-01
**ITGB1**	1.133	9.32E-01
**FUCA1**	1.133	9.34E-01
**ARAP3**	1.133	9.34E-01
**SGTA**	1.133	9.62E-01
**HLA-C**	1.133	9.74E-01
**RPL10L**	1.132	9.59E-01
**PUF60**	1.131	9.31E-01
**SCFD1**	1.131	9.28E-01
**SLTM**	1.131	9.34E-01
**SF3B1**	1.13	9.31E-01
**RBMX**	1.13	9.31E-01
**TUBB2B**	1.129	9.34E-01
**TNS1**	1.129	9.28E-01
**USP9Y**	1.129	9.34E-01
**DNAJC11**	1.128	9.34E-01
**THOC3**	1.128	9.56E-01
**NONO**	1.127	9.28E-01
**RPS17; RPS17L**	1.127	9.28E-01
**ILK**	1.127	9.28E-01
**GIGYF2**	1.127	9.31E-01
**CRTAP**	1.127	9.27E-01
**CTNNBL1**	1.127	9.58E-01
**SFPQ**	1.126	9.28E-01
**TMPO**	1.126	9.27E-01
**IVD**	1.126	9.34E-01
**KIAA1033; WASHC4**	1.126	9.62E-01
**RBBP5**	1.126	9.58E-01
**RPS29**	1.126	9.27E-01

Gene Symbol	Abundance Ratio (DMF/DMSO)	P-value
**RPL38**	1.125	9.27E-01
**MYOF**	1.124	9.28E-01
**CORO1C**	1.124	9.28E-01
**RPL23A**	1.124	9.28E-01
**POLR2B**	1.124	9.27E-01
**ARHGEF2**	1.124	9.13E-01
**FAM49A**	1.124	9.34E-01
**RPS23**	1.124	9.28E-01
**SF3B3**	1.123	9.28E-01
**DDX19B**	1.123	9.27E-01
**IDH3G**	1.123	9.26E-01
**CLUH; KIAA0664**	1.123	9.34E-01
**CYP20A1**	1.123	9.68E-01
**PGP**	1.123	9.64E-01
**PRPF6**	1.122	9.23E-01
**PPM1A**	1.122	9.34E-01
**VCL**	1.121	9.28E-01
**GPX7**	1.12	9.61E-01
**RAB11FIP1**	1.12	9.30E-01
**GSTO1**	1.12	9.53E-01
**GNB2L1; RACK1**	1.119	9.27E-01
**RPS11**	1.119	9.27E-01
**FAM129B**	1.119	9.27E-01
**UBFD1**	1.119	9.54E-01
**RRP12**	1.119	9.30E-01
**NDUFS3**	1.118	9.26E-01
**SH3BGRL3**	1.118	9.13E-01
**IGF2R**	1.118	9.29E-01
**MRPL38**	1.118	9.50E-01
**SERBP1**	1.117	9.27E-01
**STT3A**	1.117	9.27E-01
**PDS5A**	1.117	9.28E-01
**AKT3**	1.117	9.28E-01
**GOT2**	1.117	9.27E-01
**WBP11**	1.117	9.64E-01
**PCCB**	1.116	9.28E-01
**PRKCDBP; CAVIN3**	1.116	9.23E-01
**USP5**	1.115	9.27E-01
**RAP1GDS1**	1.115	9.28E-01
**MRTO4**	1.115	9.54E-01

Gene Symbol	Abundance Ratio (DMF/DMSO)	P-value
NUP188	1.115	9.83E-01
SRRM2	1.114	9.09E-01
PRKACB	1.114	9.50E-01
TOMM22	1.114	9.22E-01
RPL27A	1.114	9.22E-01
STOM	1.114	9.22E-01
STAM2	1.114	9.62E-01
PRKAA1	1.113	9.06E-01
SH3KBP1	1.112	9.28E-01
RBM15	1.112	9.62E-01
GLG1	1.111	9.13E-01
XPNPEP1	1.111	8.88E-01
RPL8	1.11	9.13E-01
NOS3	1.11	9.13E-01
RUVBL1	1.11	9.13E-01
RPL3	1.11	9.13E-01
RPL35A	1.11	9.13E-01
SPRYD4	1.11	9.72E-01
HNRNPK	1.109	9.13E-01
RPL9	1.109	9.13E-01
USP4	1.109	9.27E-01
MPP6	1.109	9.58E-01
MSN	1.108	9.10E-01
FLNC	1.108	9.31E-01
HNRNPA0	1.108	9.08E-01
UFL1	1.108	9.28E-01
PARP1	1.107	9.10E-01
TAF15	1.107	9.27E-01
ATAD1	1.106	9.53E-01
GCN1L1; GCN1	1.105	9.03E-01
U2SURP	1.105	8.97E-01
SNW1	1.105	9.23E-01
RPS13	1.104	8.99E-01
CSAD	1.104	9.26E-01
SQRDL; SQOR	1.104	9.27E-01
RHEB	1.104	9.27E-01
PLOD3	1.103	8.89E-01
CPT2	1.103	9.53E-01
ATP13A1	1.103	9.31E-01
RPL23	1.102	8.93E-01

Gene Symbol	Abundance Ratio (DMF/DMSO)	P-value
**USP10**	1.102	8.72E-01
**NPM1**	1.101	8.92E-01
**ENO3**	1.101	8.91E-01
**DDX42**	1.101	9.13E-01
**RAD21**	1.101	9.23E-01
**PES1**	1.101	9.22E-01
**RPL30**	1.1	8.90E-01
**GOLGA3**	1.1	9.21E-01
**FABP4**	1.1	8.90E-01
**MRPS27**	1.1	9.27E-01
**RPS3A**	1.099	8.89E-01
**HSPA4L**	1.099	8.80E-01
**VPS18**	1.097	9.56E-01
**RPLP2**	1.096	8.75E-01
**QDPR**	1.096	9.13E-01
**MAPKAPK2**	1.096	9.34E-01
**EIF2A**	1.095	9.10E-01
**FDPS**	1.095	8.90E-01
**SMARCB1**	1.095	9.10E-01
**TUBB4B**	1.094	8.81E-01
**PPP1R12A**	1.094	8.81E-01
**RPS14**	1.094	8.81E-01
**TXNIP; LOC101060503**	1.094	9.53E-01
**LMNB1**	1.092	8.79E-01
**RPL10**	1.092	8.80E-01
**LRRC16A; CARMIL1**	1.092	9.34E-01
**UQCRC1**	1.091	8.72E-01
**RPL34**	1.091	8.75E-01
**UFC1**	1.091	9.53E-01
**MRPL45**	1.091	9.34E-01
**FLNA**	1.09	8.75E-01
**ALDH1A1**	1.09	8.75E-01
**CDK6**	1.09	8.90E-01
**TUBA1C**	1.089	9.03E-01
**TMX3**	1.089	8.72E-01
**LEPREL4; P3H4**	1.088	9.34E-01
**GNAI2**	1.086	8.72E-01
**PDHB**	1.086	8.35E-01
**NAT2**	1.086	9.50E-01
**ARL2**	1.086	9.53E-01

Gene Symbol	Abundance Ratio (DMF/DMSO)	P-value
**PI4KA**	1.085	9.48E-01
**RPL14**	1.084	8.69E-01
**RPS21**	1.083	8.75E-01
**TIE1**	1.083	9.53E-01
**C16orf62**	1.083	9.51E-01
**FBL**	1.082	8.59E-01
**RCC2**	1.082	8.47E-01
**RCN3**	1.081	8.54E-01
**FAF1**	1.081	8.86E-01
**CRYZ**	1.081	8.88E-01
**PAFAH1B2**	1.081	8.56E-01
**RBM17**	1.081	9.13E-01
**DHCR24**	1.081	9.04E-01
**AKR1B1**	1.08	8.51E-01
**NDUFV3**	1.08	9.53E-01
**TMF1**	1.08	9.53E-01
**SNRNP70**	1.079	8.55E-01
**POFUT1**	1.079	8.82E-01
**MTCH2**	1.077	8.32E-01
**ADD1**	1.077	8.81E-01
**LSS**	1.077	8.82E-01
**NDUFA13**	1.077	8.82E-01
**ARHGAP12**	1.077	9.53E-01
**GLS**	1.076	8.51E-01
**FUS**	1.076	8.44E-01
**MTHFD1**	1.076	8.44E-01
**CCDC47**	1.076	8.75E-01
**TBC1D4**	1.075	8.09E-01
**NDUFS2**	1.074	8.29E-01
**MUT**	1.074	8.82E-01
**MRPL22**	1.074	9.34E-01
**FTSJ3**	1.073	9.34E-01
**DAG1**	1.072	9.53E-01
**RPL6**	1.071	8.28E-01
**NID2**	1.071	8.20E-01
**RPS16**	1.07	8.24E-01
**ABR**	1.07	8.74E-01
**DHX30**	1.07	8.72E-01
**CAPG**	1.069	8.58E-01
**MCM2**	1.068	8.21E-01

Gene Symbol	Abundance Ratio (DMF/DMSO)	P-value
**MYL6**	1.067	8.21E-01
**ERP29**	1.067	8.70E-01
**KIAA2013**	1.066	9.34E-01
**ALYREF**	1.065	8.14E-01
**TTLL12**	1.065	8.05E-01
**SRSF5**	1.064	8.72E-01
**MRPL43**	1.063	9.52E-01
**CLINT1**	1.062	8.09E-01
**RPL31**	1.062	8.05E-01
**UFD1L; UFD1**	1.062	8.05E-01
**WDR37**	1.062	8.79E-01
**NDUFA5**	1.062	9.56E-01
**HNRNPM**	1.061	8.04E-01
**SLC12A2**	1.061	7.92E-01
**RPS6**	1.06	8.02E-01
**RPL10A**	1.06	8.02E-01
**PRKCSH**	1.059	7.89E-01
**ASL**	1.059	8.40E-01
**LEMD3**	1.059	9.31E-01
**NDUFS8**	1.059	8.63E-01
**PCBP2**	1.058	7.99E-01
**ARHGEF1**	1.058	8.62E-01
**SULF2**	1.058	8.54E-01
**ACTC1**	1.056	7.98E-01
**BUB3**	1.055	7.60E-01
**PITRM1**	1.055	8.41E-01
**MOV10**	1.053	7.77E-01
**ACTL6A**	1.053	8.24E-01
**LNPEP**	1.053	8.24E-01
**PPIB**	1.052	7.90E-01
**PDAP1**	1.052	7.97E-01
**LRRFIP1**	1.052	9.34E-01
**NFS1**	1.051	8.51E-01
**ETFDH**	1.051	9.28E-01
**ERH**	1.051	8.12E-01
**FBLN2**	1.05	7.92E-01
**TUBGCP3**	1.05	8.48E-01
**PPIG**	1.05	9.27E-01
**CSRP1**	1.05	7.75E-01
**LEPRE1; P3H1**	1.049	7.72E-01

Gene Symbol	Abundance Ratio (DMF/DMSO)	P-value
**NEXN**	1.049	8.21E-01
**IQGAP2**	1.049	8.90E-01
**RPS28**	1.049	7.63E-01
**H1F0**	1.049	7.45E-01
**CAPN5**	1.048	7.49E-01
**PTK7**	1.048	9.28E-01
**MCM3**	1.047	7.75E-01
**MCM6**	1.047	7.75E-01
**RBBP7**	1.047	7.85E-01
**RPL35**	1.047	7.72E-01
**GALNT7**	1.047	8.45E-01
**OSGEP**	1.047	9.03E-01
**THOP1**	1.046	8.16E-01
**GBAS; NIPSNAP2**	1.046	7.35E-01
**PGRMC2**	1.046	7.35E-01
**LARS2**	1.046	8.88E-01
**MFAP1**	1.046	9.30E-01
**CTNNA1**	1.045	7.65E-01
**HIST1H2AB; HIST1H2AE**	1.045	7.31E-01
**RBBP4**	1.045	8.02E-01
**APPL2**	1.045	8.06E-01
**SNRPB2**	1.045	8.72E-01
**KANK3**	1.045	9.28E-01
**HEXA**	1.044	9.52E-01
**UBTF**	1.043	8.23E-01
**PPM1F**	1.043	8.10E-01
**VAMP8**	1.043	9.34E-01
**MCM5**	1.042	7.54E-01
**MTDH**	1.042	7.55E-01
**CCAR1**	1.042	8.09E-01
**CDC40**	1.042	9.34E-01
**MOGS**	1.042	8.99E-01
**DOCK6**	1.041	8.02E-01
**PLCB3**	1.041	7.99E-01
**EPN2**	1.041	9.34E-01
**ASPSCR1**	1.041	9.34E-01
**ARFGAP2**	1.04	7.51E-01
**MAT2A**	1.039	7.37E-01
**MRPS34**	1.039	8.28E-01
**GALNT1**	1.038	8.05E-01

Gene Symbol	Abundance Ratio (DMF/DMSO)	P-value
**MAP2K2**	1.038	8.09E-01
**ACADSB**	1.038	8.14E-01
**RPL4**	1.037	7.35E-01
**RPL7**	1.037	7.37E-01
**GBF1**	1.037	7.90E-01
**NUP98**	1.037	8.05E-01
**TUBB6**	1.036	7.35E-01
**RPL18**	1.036	7.35E-01
**ALDH1A2**	1.034	7.31E-01
**MAPK3**	1.034	7.32E-01
**SDPR; CAVIN2**	1.034	7.22E-01
**LTA4H**	1.034	7.99E-01
**SLC25A11**	1.034	7.24E-01
**PUS7**	1.034	9.28E-01
**HNRNPL**	1.033	7.31E-01
**RPL5**	1.033	7.29E-01
**STAG2**	1.033	8.09E-01
**CDH13**	1.032	7.26E-01
**DEK**	1.032	7.17E-01
**CALU**	1.031	8.03E-01
**MCM4**	1.03	7.23E-01
**IPO9**	1.03	8.16E-01
**PPT1**	1.03	8.09E-01
**MYH11**	1.029	7.31E-01
**ARL3**	1.029	7.99E-01
**SARNP**	1.029	8.02E-01
**APOOL**	1.029	8.40E-01
**FASN**	1.028	7.19E-01
**SNRPD1**	1.028	7.22E-01
**HIP1**	1.028	7.55E-01
**ZMPSTE24**	1.028	9.34E-01
**TLN1**	1.027	7.17E-01
**TUBG1**	1.027	7.17E-01
**SMU1**	1.027	6.97E-01
**PTGS1**	1.027	8.02E-01
**DDX27**	1.027	8.72E-01
**CDK5**	1.027	9.31E-01
**RPL7A**	1.026	7.12E-01
**TPM1**	1.026	9.34E-01
**ACADS**	1.026	7.32E-01

Gene Symbol	Abundance Ratio (DMF/DMSO)	P-value
**PCBP1**	1.024	7.07E-01
**RPL13A**	1.023	7.02E-01
**PGM3**	1.023	7.04E-01
**RABEP2**	1.023	7.58E-01
**SCRIB**	1.023	9.34E-01
**RPS7**	1.022	7.01E-01
**UCHL3**	1.022	7.85E-01
**ABLIM1**	1.022	9.01E-01
**RAD23B**	1.021	6.64E-01
**PRDX3**	1.021	7.73E-01
**CYR61**	1.02	6.98E-01
**AIMP2**	1.02	7.98E-01
**ZW10**	1.019	8.95E-01
**MYL12B; MYL12A**	1.018	6.83E-01
**MYBBP1A**	1.017	6.82E-01
**TIMM50**	1.016	7.31E-01
**SMC4**	1.016	7.63E-01
**RASIP1**	1.015	6.76E-01
**SMC1A**	1.015	6.51E-01
**NAT10**	1.015	7.45E-01
**GMPPA**	1.015	7.92E-01
**RPL27**	1.014	6.67E-01
**MCM7**	1.013	6.64E-01
**FUBP1**	1.013	6.65E-01
**PRPF31**	1.013	7.39E-01
**GTPBP1**	1.013	9.28E-01
**STX12**	1.013	9.28E-01
**PAPOLA**	1.013	8.93E-01
**PCCA**	1.012	7.71E-01
**CTNNB1**	1.01	6.53E-01
**TECR**	1.01	6.76E-01
**EPN1**	1.01	7.39E-01
**MPC2**	1.01	7.35E-01
**RPS24**	1.009	6.53E-01
**SNRPA**	1.009	7.55E-01
**RPF2**	1.008	7.35E-01
**SRM**	1.008	6.43E-01
**ACTG1**	1.005	6.37E-01
**RBM12B**	1.005	8.33E-01
**FNTA**	1.004	8.23E-01

Gene Symbol	Abundance Ratio (DMF/DMSO)	P-value
GPX4	1.004	6.11E-01
DCP1A	1.003	8.82E-01
PDIA5	1.001	7.58E-01
EEF2	1	6.19E-01
CHD4	1	5.86E-01
RRAS	1	7.09E-01
NEMF	1	9.26E-01
SRC	0.999	9.31E-01
BYSL	0.999	9.34E-01
CEP290	0.999	7.35E-01
JUP	0.998	5.88E-01
ARRB1	0.998	7.09E-01
CNBP	0.998	7.35E-01
MTA1	0.998	9.34E-01
FBLIM1	0.998	7.26E-01
DDX31	0.997	5.86E-01
CUL5	0.997	9.19E-01
SMC3	0.996	6.16E-01
CCAR2; KIAA1967	0.996	5.98E-01
SKIV2L	0.996	7.21E-01
SRPR; SRPRA	0.995	5.98E-01
RPL19	0.995	5.99E-01
STAB1	0.995	8.72E-01
CNOT2	0.995	8.24E-01
HNRNPU	0.994	5.98E-01
PGM5	0.994	5.98E-01
RPL13	0.994	5.97E-01
QRICH1	0.994	8.70E-01
EHD3	0.993	7.17E-01
MRPL19	0.992	9.27E-01
CTSA	0.991	7.19E-01
PELP1	0.991	8.29E-01
GPX8	0.991	8.82E-01
PUS1	0.99	9.22E-01
SF3B2	0.989	5.86E-01
EEF1A2	0.989	7.02E-01
PICALM	0.988	5.86E-01
NEK7	0.988	8.14E-01
RPL32	0.987	5.86E-01
CHMP1A	0.987	8.91E-01

Gene Symbol	Abundance Ratio (DMF/DMSO)	P-value
**RPL26**	0.986	5.86E-01
**SMARCA2**	0.986	9.27E-01
**RPL39; RPL39P3**	0.986	5.60E-01
**PPIA**	0.985	5.82E-01
**GANAB**	0.984	5.82E-01
**ACAD9**	0.983	8.89E-01
**CPOX**	0.982	6.57E-01
**NUP133**	0.982	7.16E-01
**MRPL2**	0.981	6.97E-01
**BCLAF1**	0.98	5.80E-01
**RPL37A**	0.979	5.44E-01
**MPZL1**	0.978	8.93E-01
**GNB4**	0.977	6.30E-01
**PSME1**	0.977	5.60E-01
**LUC7L**	0.977	8.47E-01
**PTGIS**	0.977	5.61E-01
**PTMA**	0.977	6.64E-01
**RBM10**	0.976	7.89E-01
**ZFPL1**	0.975	8.15E-01
**HP1BP3**	0.974	5.50E-01
**TBCD**	0.974	6.86E-01
**AKT1**	0.974	8.82E-01
**CHD3**	0.973	9.28E-01
**ARHGAP29**	0.973	9.15E-01
**DNPEP**	0.971	5.86E-01
**RPL24**	0.971	5.31E-01
**HIST1H1B**	0.97	5.23E-01
**WDR5**	0.97	8.46E-01
**SPTLC1**	0.968	5.16E-01
**TPM3**	0.967	5.13E-01
**PNPT1**	0.967	6.82E-01
**MYH14**	0.966	9.28E-01
**HDGFRP3; HDGFL3**	0.966	9.10E-01
**SART1**	0.965	6.12E-01
**S100A4**	0.965	5.06E-01
**SMAP2**	0.965	6.12E-01
**TOM1**	0.965	7.23E-01
**ACTN1**	0.964	8.81E-01
**HMGB2**	0.964	4.67E-01
**ABCE1**	0.964	5.15E-01

Gene Symbol	Abundance Ratio (DMF/DMSO)	P-value
**SORBS2**	0.962	4.91E-01
**SNRPB**	0.962	4.39E-01
**TIMP3**	0.962	5.70E-01
**HEXB**	0.962	8.80E-01
**CASP3**	0.961	6.53E-01
**BAZ1B**	0.961	8.75E-01
**THRAP3**	0.96	4.91E-01
**NBAS**	0.96	6.38E-01
**GGCX**	0.96	6.19E-01
**CDK1**	0.959	4.91E-01
**DOCK1**	0.959	7.10E-01
**P4HA2**	0.958	4.78E-01
**RPL17-C18ORF32; RPL17**	0.957	4.72E-01
**KRT10**	0.956	4.67E-01
**RBM27**	0.956	9.28E-01
**HNRNPH1**	0.955	4.84E-01
**MYH9**	0.954	4.60E-01
**PDLIM5**	0.954	4.62E-01
**GTPBP4**	0.954	5.92E-01
**CUX1**	0.954	8.82E-01
**FKBP10**	0.953	5.82E-01
**STAT5A**	0.953	5.97E-01
**SMC2**	0.952	5.82E-01
**DPYSL3**	0.951	4.51E-01
**NUCB1**	0.95	4.59E-01
**TNS3**	0.949	8.50E-01
**HMGB1**	0.948	4.34E-01
**EBNA1BP2**	0.947	5.86E-01
**PNO1**	0.947	8.72E-01
**ERLIN2**	0.944	4.14E-01
**KRT9**	0.943	4.18E-01
**DHCR7**	0.943	6.74E-01
**COL4A1**	0.942	5.86E-01
**TENC1; TNS2**	0.942	4.26E-01
**GFM1**	0.941	4.26E-01
**PFDN6**	0.94	8.93E-01
**B3GAT3**	0.939	8.50E-01
**CORO7-PAM16; CORO7**	0.939	5.91E-01
**SELENBP1**	0.938	3.72E-01
**HIST2H2BF**	0.935	3.86E-01

Gene Symbol	Abundance Ratio (DMF/DMSO)	P-value
**FHL3**	0.935	5.72E-01
**FAM98B**	0.934	8.40E-01
**WDR61**	0.934	5.72E-01
**POLR1A**	0.934	8.77E-01
**BRD4**	0.934	8.48E-01
**KDM1A**	0.93	5.50E-01
**POLR1C**	0.928	6.10E-01
**CTSK**	0.928	5.71E-01
**STAT6**	0.928	8.28E-01
**ANXA11**	0.927	5.29E-01
**PURB**	0.927	7.62E-01
**PDP1**	0.927	6.87E-01
**POTEI**	0.926	4.91E-01
**ESYT1**	0.925	3.51E-01
**UROD**	0.924	5.16E-01
**POTEE**	0.923	3.47E-01
**OAT**	0.923	3.61E-01
**SUN2**	0.923	3.68E-01
**ADAR**	0.923	3.61E-01
**CBX5**	0.923	5.47E-01
**TAGLN**	0.919	3.33E-01
**ACTBL2**	0.913	3.12E-01
**CTNND1**	0.912	3.16E-01
**HIST1H2BO**	0.911	3.28E-01
**MAN1A1**	0.911	8.21E-01
**CCNYL1**	0.91	6.51E-01
**SACS**	0.909	3.47E-01
**AP1S1**	0.907	5.82E-01
**SKIV2L2**	0.906	3.42E-01
**ADPRHL2**	0.906	7.19E-01
**SPATA20**	0.904	3.00E-01
**HAT1**	0.904	5.10E-01
**PDK3**	0.902	8.02E-01
**NDUFS1**	0.901	2.96E-01
**LMCD1**	0.901	2.75E-01
**SMEK1; PPP4R3A**	0.9	7.35E-01
**PML**	0.9	7.23E-01
**LSM4**	0.898	4.90E-01
**DENND4C**	0.897	4.26E-01
**ABHD2**	0.897	3.80E-01

Gene Symbol	Abundance Ratio (DMF/DMSO)	P-value
**ASCC3**	0.896	8.02E-01
**HIST1H1D**	0.894	2.73E-01
**KRT2**	0.893	2.46E-01
**RAD23A**	0.892	7.02E-01
**PRKACA**	0.891	3.86E-01
**PDLIM7**	0.891	2.91E-01
**NSUN2**	0.888	4.53E-01
**NDUFV1**	0.887	2.37E-01
**CNN3**	0.884	2.39E-01
**PPP1R13L**	0.884	5.10E-01
**IKBKAP; ELP1**	0.884	5.51E-01
**NEFM**	0.883	7.57E-01
**PDPR**	0.883	4.18E-01
**NDUFA4**	0.883	2.73E-01
**DHFRL1; DHFR2**	0.883	5.86E-01
**RRM1**	0.882	2.47E-01
**PRDX2**	0.882	2.41E-01
**RPL21**	0.881	2.34E-01
**PPP2R5C**	0.881	7.99E-01
**LRP1**	0.881	7.99E-01
**SYNPO**	0.88	2.65E-01
**TXNDC5**	0.879	2.34E-01
**CIRBP**	0.879	6.06E-01
**CAMK1D**	0.878	7.04E-01
**FDFT1**	0.877	3.89E-01
**KRT1**	0.871	2.09E-01
**PFDN2**	0.871	2.34E-01
**AHCYL1**	0.867	3.86E-01
**RAB11A**	0.867	7.39E-01
**NPEPL1**	0.867	6.63E-01
**DSTN**	0.866	1.95E-01
**ABCA7**	0.866	7.41E-01
**VAC14**	0.863	7.99E-01
**HDAC2**	0.861	7.45E-01
**LEPREL2; P3H3**	0.86	3.49E-01
**TUBB**	0.858	1.74E-01
**FNBP1**	0.858	2.79E-01
**NFKB1**	0.857	3.50E-01
**HLA-C**	0.857	1.74E-01
**FABP5**	0.856	1.56E-01

Gene Symbol	Abundance Ratio (DMF/DMSO)	P-value
**FAR1**	0.856	3.14E-01
**HLA-C**	0.855	3.16E-01
**PHLDB1**	0.853	6.23E-01
**FRY**	0.849	7.26E-01
**SGCD**	0.848	4.99E-01
**FN1**	0.847	1.50E-01
**PBXIP1**	0.847	3.16E-01
**PPIL1**	0.847	3.16E-01
**MRI1**	0.845	5.15E-01
**NTN4**	0.845	3.16E-01
**FNDC3B**	0.842	2.34E-01
**DDAH1**	0.84	1.33E-01
**GRWD1**	0.839	6.65E-01
**NCAPG**	0.836	5.88E-01
**CHMP2A**	0.834	5.20E-01
**ARFGEF1**	0.833	7.62E-01
**NXN**	0.832	5.88E-01
**MTOR**	0.831	5.86E-01
**NTAN1**	0.831	6.38E-01
**SYNE1**	0.83	1.23E-01
**WIBG; PYM1**	0.83	6.60E-01
**ENO2**	0.827	7.14E-01
**MRPL15**	0.825	2.58E-01
**XDH**	0.824	1.47E-01
**TMSB4X**	0.823	1.23E-01
**MTA2**	0.82	1.11E-01
**IGFBP7**	0.818	2.78E-01
**ELMO1**	0.816	6.19E-01
**DBNL**	0.815	2.16E-01
**OGT**	0.815	3.16E-01
**PDE4A**	0.815	6.19E-01
**RHOB**	0.814	2.09E-01
**ABHD16A**	0.814	5.63E-01
**HSPG2**	0.812	8.61E-02
**SMAD3**	0.812	5.10E-01
**FOXK1**	0.812	5.86E-01
**DDB1**	0.811	8.41E-02
**BRIX1**	0.811	5.25E-01
**STMN1**	0.806	1.54E-01
**NCAPD2**	0.804	6.47E-01

Gene Symbol	Abundance Ratio (DMF/DMSO)	P-value
**FEN1**	0.802	2.31E-01
**RAVER1**	0.8	1.89E-01
**TGFB1I1**	0.8	4.55E-01
**ANKLE2**	0.799	4.67E-01
**EEFSEC**	0.798	5.88E-01
**CALD1**	0.797	6.37E-02
**CELF2**	0.796	5.98E-01
**UBE2D2**	0.795	6.79E-01
**TPM4**	0.794	5.96E-02
**ISYNA1**	0.792	5.80E-01
**RBM45**	0.791	1.54E-01
**ITGA3**	0.788	1.87E-01
**H1FX**	0.787	4.82E-02
**C3orf58**	0.786	4.95E-02
**LSG1**	0.785	1.96E-01
**FAF2**	0.784	5.86E-01
**MSH6**	0.782	1.07E-01
**GTF2I**	0.781	4.03E-02
**ZRANB2**	0.779	1.47E-01
**LLGL1**	0.778	5.99E-01
**PKN1**	0.775	5.82E-01
**WNT5A**	0.774	3.16E-01
**TUBB3**	0.769	3.55E-02
**RFC5**	0.761	4.72E-01
**CDR2L**	0.757	5.86E-01
**COL18A1**	0.751	2.22E-02
**KDELR1**	0.749	4.67E-01
**DNMT1**	0.749	1.14E-01
**EPM2AIP1**	0.748	1.09E-01
**PECAM1**	0.748	1.21E-01
**GNA14**	0.746	4.99E-01
**VKORC1L1**	0.742	3.58E-01
**THBS2**	0.741	5.86E-01
**ERG**	0.74	2.58E-02
**SPECC1L**	0.736	4.07E-01
**PALMD**	0.735	2.46E-02
**BCKDHA**	0.729	6.38E-02
**ZYX**	0.727	2.19E-02
**MMS19**	0.726	8.91E-02
**GLS**	0.722	8.41E-02

Gene Symbol	Abundance Ratio (DMF/DMSO)	P-value
**FILIP1L**	0.718	9.90E-03
**PRUNE2**	0.717	1.30E-01
**HDAC1**	0.711	3.69E-02
**GATAD2B**	0.711	6.12E-02
**NUP85**	0.711	3.42E-01
**INF2**	0.699	3.55E-01
**MSH2**	0.697	2.66E-02
**SPECC1**	0.691	1.26E-01
**NDUFS7**	0.691	9.03E-02
**PCDH7**	0.69	2.68E-01
**DMD**	0.688	3.27E-01
**DDX54**	0.679	4.59E-01
**ELTD1; ADGRL4**	0.674	1.78E-01
**TPM4**	0.673	3.16E-01
**PTPRM**	0.663	1.30E-02
**SH3BP4**	0.662	1.61E-01
**ABCF3**	0.66	1.54E-01
**NFKB2**	0.657	3.30E-02
**UFM1**	0.654	2.73E-01
**AOX1**	0.652	1.92E-03
**ERAP1**	0.652	1.86E-02
**SMAD1**	0.651	2.41E-01
**CSRP2**	0.646	9.15E-03
**EML3**	0.64	2.47E-01
**HID1**	0.635	3.30E-03
**FBLN5**	0.627	1.36E-02
**ZSWIM8**	0.623	3.27E-01
**BTF3**	0.605	2.68E-02
**VCAN**	0.599	1.58E-01
**CGNL1**	0.586	2.32E-04
**PDCD4**	0.585	1.09E-04
**COL15A1**	0.583	4.53E-03
**CNN2**	0.581	8.27E-05
**SPARC**	0.577	4.13E-04
**TLE1**	0.569	7.83E-03
**COL5A2**	0.553	1.75E-03
**CSNK1E; LOC400927-CSNK1E**	0.548	1.24E-01
**GDF6**	0.546	3.88E-03
**PLXND1**	0.544	6.88E-02
**KCTD12**	0.521	1.17E-06

Gene Symbol	Abundance Ratio (DMF/DMSO)	P-value
**COL14A1**	0.51	1.85E-06
**CRIM1**	0.507	6.12E-02
**COL3A1**	0.496	2.95E-05
**KPNA2**	0.486	2.77E-07
**YAP1**	0.482	3.75E-02
**SARDH**	0.479	1.12E-02
**BRD3**	0.467	3.69E-02
**FSTL1**	0.456	8.44E-06
**COL5A1**	0.45	2.51E-06
**CTGF**	0.444	2.02E-09
**BGN**	0.436	6.43E-05
**VCAN**	0.432	3.41E-02
**EEF2K; LOC101930123**	0.394	7.90E-07
**GPRC5B**	0.387	2.76E-08
**ACO1**	0.353	8.59E-14
**FBLN1**	0.349	2.26E-14
**CXCR4**	0.336	1.61E-05
**WWTR1**	0.248	7.16E-06
**COL12A1**	0.225	1.03E-15
**SERPINE1**	0.206	1.03E-15
**TPM1**	0.135	1.03E-15
**THBS1**	0.133	1.03E-15
**TPM1**	0.106	1.03E-15

Supplementary Table S2. Proteins detected with the proteomics analysis in HMECs with an abundance ratio (DMF/DMSO) > 1.5 and p-value < 0.01.

Gene Symbol	Abundance Ratio (DMF/DMSO)	P-value
**HMOX1**	26.203	1.03E-15
**GCLC**	7.12	1.03E-15
**ABHD4**	7.001	1.53E-05
**GBE1**	4.463	1.72E-11
**PSPH**	4.461	3.11E-11
**ARHGAP24**	4.429	3.81E-04
**SLC2A14**	4.063	7.13E-06
**DNM1**	3.912	3.82E-03
**TXNRD1**	3.904	2.96E-11
**SLC2A1**	3.72	7.95E-03
**PCYT1A**	3.526	5.65E-05
**HSPA1B; HSPA1A**	3.497	2.73E-09
**DCD**	3.446	6.15E-06
**VTN**	3.359	9.10E-04
**VWA5A**	3.236	2.94E-04
**ASNS**	3.117	2.83E-07
**RTN4**	3.107	6.39E-06
**SQSTM1**	3.04	5.74E-04
**ALDH1L2**	3.012	2.51E-05
**HSPH1**	2.991	1.27E-06
**BAG3**	2.932	4.46E-03
**CHORDC1**	2.765	1.16E-03
**DNAJA1**	2.76	6.39E-05
**SHANK3**	2.755	5.95E-03
**ITGA2**	2.752	8.27E-05
**SARS**	2.519	6.21E-04
**CACYBP**	2.516	1.62E-03
**HSPB1**	2.51	2.97E-04
**CLIP1**	2.496	8.65E-03
**PTGES3**	2.445	1.62E-03
**TXN**	2.34	1.87E-03
**COTL1**	2.332	8.80E-03
**HBD**	2.324	4.52E-03
**FKBP5**	2.288	6.38E-03
**SNTB2**	2.282	7.00E-03
**SNX3**	2.189	9.15E-03
**HECTD1**	2.183	8.80E-03

Supplementary Table S3. Proteins detected with the proteomics analysis in HMECs with an abundance ratio (DMF/DMSO) < 0.75 and p-value < 0.01.

Gene Symbol	Abundance Ratio (DMF/DMSO)	P-value
**TPM1**	0.106	1.03E-15
**THBS1**	0.133	1.03E-15
**TPM1**	0.135	1.03E-15
**SERPINE1**	0.206	1.03E-15
**COL12A1**	0.225	1.03E-15
**WWTR1**	0.248	7.16E-06
**CXCR4**	0.336	1.61E-05
**FBLN1**	0.349	2.26E-14
**ACO1**	0.353	8.59E-14
**GPRC5B**	0.387	2.76E-08
**EEF2K; LOC101930123**	0.394	7.90E-07
**BGN**	0.436	6.43E-05
**CTGF**	0.444	2.02E-09
**COL5A1**	0.45	2.51E-06
**FSTL1**	0.456	8.44E-06
**KPNA2**	0.486	2.77E-07
**COL3A1**	0.496	2.95E-05
**COL14A1**	0.51	1.85E-06
**KCTD12**	0.521	1.17E-06
**GDF6**	0.546	3.88E-03
**COL5A2**	0.553	1.75E-03
**TLE1**	0.569	7.83E-03
**SPARC**	0.577	4.13E-04
**CNN2**	0.581	8.27E-05
**COL15A1**	0.583	4.53E-03
**PDCD4**	0.585	1.09E-04
**CGNL1**	0.586	2.32E-04
**HID1**	0.635	3.30E-03
**CSRP2**	0.646	9.15E-03
**AOX1**	0.652	1.92E-03
**FILIP1L**	0.718	9.90E-03
