# Supplementary material for: New insights in the targets of action of dimethyl fumarate in endothelial cells: effects on energetic metabolism and serine synthesis in vitro and in vivo
Source: Commun Biol. 2023 Oct 25;6:1084. doi: 10.1038/s42003-023-05443-4 (PMC10600195; doi:10.1038/s42003-023-05443-4)
Supplement: Supplementary file 3 — Description of Additional Supplementary Files [file 42003_2023_5443_MOESM3_ESM.pdf]

Description of Additional Supplementary Files

File name: Supplementary Data 1
Description: XLSX file, including peak area from GC/MS experiment corresponding to data from Supplementary Figure 7.

File name: Supplementary Data 2
Description: XLSX file, including peak area from GC/MS experiment corresponding to data from Figures 2 and 3 and Supplementary Figure 6.

File name: Supplementary Data 3
Description: XLSX file, including peak area from GC/MS experiment corresponding to data from Figure 4.

File name: Supplementary Data 4
Description: XLSX file, including peak area from GC/MS experiment corresponding to data from Figures 5B, 5C and 5F.

File name: Supplementary Data 5
Description: XLSX file, including peak area from GC/MS experiment corresponding to data from Figures 5D, 5E and 5G.

File name: Supplementary Data 6
Description: XLSX file, including peak area from GC/MS experiment corresponding to data from Supplementary Figure 8.

File name: Supplementary Data 7
Description: XLSX file, including peak area from positive mode LC/MS experiment corresponding to data from Supplementary Figure 6.

File name: Supplementary Data 8
Description: XLSX file, including peak area from negative mode LC/MS experiment corresponding to data from Supplementary Figure 6.
