# Supplementary material for: New insights in the targets of action of dimethyl fumarate in endothelial cells: effects on energetic metabolism and serine synthesis in vitro and in vivo
Source: Commun Biol. 2023 Oct 25;6:1084. doi: 10.1038/s42003-023-05443-4 (PMC10600195; doi:10.1038/s42003-023-05443-4)
Supplement: Supplementary file 12 — Reporting Summary [file 42003_2023_5443_MOESM12_ESM.pdf]

nature portfolio

Corresponding author(s): Miguel Ángel Medina Torres & Beatriz Martínez-Poveda

Last updated by author(s): 2023/09/11

Reporting Summary

Nature Portfolio wishes to improve the reproducibility of the work that we publish. This form provides structure for consistency and transparency in reporting. For further information on Nature Portfolio policies, see our Editorial Policies and the Editorial Policy Checklist.

Statistics

For all statistical analyses, confirm that the following items are present in the figure legend, table legend, main text, or Methods section.

n/a	Confirmed	
☐	☒	The exact sample size (*n*) for each experimental group/condition, given as a discrete number and unit of measurement
☐	☒	A statement on whether measurements were taken from distinct samples or whether the same sample was measured repeatedly
☐	☒	The statistical test(s) used AND whether they are one- or two-sided<br>*Only common tests should be described solely by name; describe more complex techniques in the Methods section.*
☐	☒	A description of all covariates tested
☒	☐	A description of any assumptions or corrections, such as tests of normality and adjustment for multiple comparisons
☐	☒	A full description of the statistical parameters including central tendency (e.g. means) or other basic estimates (e.g. regression coefficient) AND variation (e.g. standard deviation) or associated estimates of uncertainty (e.g. confidence intervals)
☐	☒	For null hypothesis testing, the test statistic (e.g. F, t, r) with confidence intervals, effect sizes, degrees of freedom and P value noted<br>*Give P values as exact values whenever suitable.*
☒	☐	For Bayesian analysis, information on the choice of priors and Markov chain Monte Carlo settings
☒	☐	For hierarchical and complex designs, identification of the appropriate level for tests and full reporting of outcomes
☒	☐	Estimates of effect sizes (e.g. Cohen's d, Pearson's r), indicating how they were calculated

Our web collection on statistics for biologists contains articles on many of the points above.

Software and code

Policy information about availability of computer code

Data collection	Image Lab (Bio-Rad); MSD ChemStation (Agilent); Profinder B.08.00 SP3 (Agilent); CHENOMX 8.3 (CHENOMX Inc. Edmont CA, USA); Advance 3 Console (Bruker BioSpin, EttlinGen, Germany); Olympus Slideview VS200 Digital Slide Scanner (Olympus)
Data analysis	BD FACSuite (BD Biosciences); Image J (NIH); Proteome Discoverer 2.2 (Thermo Fisher Scientific); Heatmapper (Babicki et al., 2016, DOI 10.1093/nar/gkw419); Wave 2.6.1 (Agilent)

For manuscripts utilizing custom algorithms or software that are central to the research but not yet described in published literature, software must be made available to editors and reviewers. We strongly encourage code deposition in a community repository (e.g. GitHub). See the Nature Portfolio guidelines for submitting code & software for further information.

Data

Policy information about availability of data

All manuscripts must include a data availability statement. This statement should provide the following information, where applicable:

- Accession codes, unique identifiers, or web links for publicly available datasets
- A description of any restrictions on data availability
- For clinical datasets or third party data, please ensure that the statement adheres to our policy

Provide your data availability statement here.

Field-specific reporting

Please select the one below that is the best fit for your research. If you are not sure, read the appropriate sections before making your selection.

☒ Life sciences ☐ Behavioural & social sciences ☐ Ecological, evolutionary & environmental sciences

For a reference copy of the document with all sections, see nature.com/documents/nr-reporting-summary-flat.pdf

Life sciences study design

All studies must disclose on these points even when the disclosure is negative.

Sample size	No sample-size calculation was performed, sample-sizes were determined based in a minimal number of experimental observations which make possible to perform statistical analysis. Of note, this is not a clinical trial or clinic study.
Data exclusions	No data were excluded from the analysis.
Replication	Results from 1H HR-MAS NMR are expressed as the data obtained for the sum of all the fin sections pooled for each experimental condition. All other results are expressed as means ± SD. Data shown for extracellular flux analysis are for a representative experiment with 3 replicates, which was repeated three times. Metabolomics and labeling experiments were performed once with 3 replicates for GCMS data and 4 replicates for LCMS data. Data in the remaining figure panels reflect three independent experiments unless otherwise specified.
Randomization	In cell culture studies included in the manuscript, randomization was not applied, since samples are derived from cell cultures grown in the same environmental conditions and subjected to the same variables, only differentiating in the performed treatment. In in vivo experiments, randomization has been applied as described in Materials and Methods section.
Blinding	Blinding was not relevant for our study, since all the collected results are quantitatively assessed.

Reporting for specific materials, systems and methods

We require information from authors about some types of materials, experimental systems and methods used in many studies. Here, indicate whether each material, system or method listed is relevant to your study. If you are not sure if a list item applies to your research, read the appropriate section before selecting a response.

Materials & experimental systems

n/a	Involved in the study	
☐	☒	Antibodies
☐	☒	Eukaryotic cell lines
☒	☐	Palaeontology and archaeology
☐	☒	Animals and other organisms
☒	☐	Human research participants
☒	☐	Clinical data
☒	☐	Dual use research of concern

Methods

n/a	Involved in the study	
☒	☐	ChIP-seq
☒	☐	Flow cytometry
☒	☐	MRI-based neuroimaging

Antibodies

Antibodies used	(1) Mouse Anti-Human HIF1α (aa. 610-727) monoclonal antibody (Clone 54/HIF-1a, cat. number 610958) was from BD Biosciences (San Jose, CA, USA).<br>(2) Mouse Anti-α-tubulin monoclonal antibody (DM1A; #3873) was from Cell Signaling Technology (Danvers, MA, USA).<br>(3) Rabbit Anti-PHGDH polyclonal antibody (cat. number CTX64499) was from GeneTex (Irvine, CA, USA).<br>(4) Rabbit Anti-calnexin polyclonal antibody was from Enzo Life Sciences (Farmingdale, NY, USA).
Validation	(1) https://www.bdbiosciences.com/en-es/products/reagents/microscopy-imaging-reagents/immunofluorescence-reagents/purified-mouse-anti-human-hif-1.610958<br>(2) https://www.cellsignal.com/products/primary-antibodies/a-tubulin-dm1a-mouse-mab/3873<br>(3) https://www.genetex.com/Product/Detail/PHGDH-antibody/GTX64499<br>(4) https://www.enzolifesciences.com/ADI-SPA-860/calnexin-polyclonal-antibody/

Eukaryotic cell lines

Policy information about cell lines

Cell line source(s)	Human microvascular endothelial cells (HMECs) were kindly supplied by Dr. Arjan W. Griffioen (Maastricht University, Netherlands).

	Human umbilical vein endothelial cells (HUVECs) were isolated by a modified collagenase treatment as previously reported (Kubota, Kleinman, Martin, & Lawley, 1988).<br>Bovine aortic endothelial cells (BAECs) were isolated from bovine aortic arches as previously described and maintained in (Cardenas, Quesada, & Medina, 2006).<br>Primary human gingival fibroblasts (HGF) were adquired from ATCC (https://www.atcc.org/products/pcs-201-018).<br>Human breast carcinoma MDA-MB-231 and MCF7, human cervix adenocarcinoma HeLa were purchased from the ATCC (Rockville, MD, USA).
Authentication	HMECs: https://pubmed.ncbi.nlm.nih.gov/1361507/; CD31 detected by flow cytometry.<br>HUVECs: primary cultures were validated by flow cytometry for CD31 expression.<br>HGF: https://www.atcc.org/products/pcs-201-018<br>MDA-MB-231: https://www.atcc.org/products/crm-htb-26<br>MCF-7: https://www.atcc.org/products/htb-22
Mycoplasma contamination	Negative
Commonly misidentified lines<br>(See ICLAC register)	*Name any commonly misidentified cell lines used in the study and provide a rationale for their use.*

Animals and other organisms

Policy information about studies involving animals; ARRIVE guidelines recommended for reporting animal research

Laboratory animals	Zebrafish (Danio rerio) wildtype genotype
Wild animals	*Provide details on animals observed in or captured in the field; report species, sex and age where possible. Describe how animals were caught and transported and what happened to captive animals after the study (if killed, explain why and describe method; if released, say where and when) OR state that the study did not involve wild animals.*
Field-collected samples	*For laboratory work with field-collected samples, describe all relevant parameters such as housing, maintenance, temperature, photoperiod and end-of-experiment protocol OR state that the study did not involve samples collected from the field.*
Ethics oversight	Animal experiments were performed in accordance with the Spanish and European Guidelines for Care and Use of Laboratory Animals (R.D. 53/2013 and 2010/62/UE) and approved by the Animal Ethics Committee of the University of Málaga, and the Highest Institutional Ethical Committee (Andalusian Government, accreditation number 04/05/2021/069).

Note that full information on the approval of the study protocol must also be provided in the manuscript.
